# QTL Mapping of Fiber-Related Traits Based on a High-Density Genetic Map in Flax (*Linum usitatissimum* L.)

**DOI:** 10.3389/fpls.2018.00885

**Published:** 2018-07-17

**Authors:** Jianzhong Wu, Qian Zhao, Liyan Zhang, Suiyan Li, Yanhua Ma, Liyan Pan, Hong Lin, Guangwen Wu, Hongmei Yuan, Ying Yu, Xun Wang, Xue Yang, Zhugang Li, Tingbo Jiang, Dequan Sun

**Affiliations:** ^1^Institute of Forage and Grassland Sciences, Heilongjiang Academy of Agricultural Sciences, Harbin, China; ^2^State Key Laboratory of Tree Genetics and Breeding, Northeast Forestry University, Harbin, China; ^3^Heilongjiang Academy of Agricultural Sciences, Harbin, China

**Keywords:** *Linum usitatissimum* L., specific-locus amplified fragment (SLAF), high-density genetic map, quantitative trait loci (QTL), flax

## Abstract

A genetic map is an important and valuable tool for quantitative trait locus (QTL) mapping, marker-assisted selection (MAS)-based breeding, and reference-assisted chromosome assembly. In this study, 112 F_2_ plants from a cross between *Linum usitatissimum* L. “DIANE” and “NY17” and parent plants were subjected to high-throughput sequencing and specific-locus amplified fragment (SLAF) library construction. After preprocessing, 61.64 Gb of raw data containing 253.71 Mb paired-end reads, each 101 bp in length, were obtained. A total of 192,797 SLAFs were identified, of which 23,115 were polymorphic, with a polymorphism rate of 11.99%. Finally, 2,339 SLAFs were organized into a linkage map consisting of 15 linkage groups (LGs). The total length of the genetic map was 1483.25 centimorgans (cM) and the average distance between adjacent markers was 0.63 cM. Combined with flax chromosome-scale pseudomolecules, 12 QTLs associating with 6 flax fiber-related traits were mapped on the chromosomal scaffolds. This high-density genetic map of flax should serve as a foundation for flax fine QTL mapping, draft genome assembly, and MAS-guided breeding. Ultimately, the genomic regions identified in this research could potentially be valuable for improving flax fiber cultivars, as well as for identification of candidate genes involved in flax fiber formation processes.

**Significance statement:**

A high-density genetic map of flax was constructed, and QTLs were identified on the sequence scaffolds to be interrelated with fiber-related traits. The results of this study will not only provide a platform for gene/QTL fine mapping, map-based gene isolation, and molecular breeding for flax, but also provide a reference to help position sequence scaffolds on the physical map and assist in the process of assembling the flax genome sequence.

## Introduction

Flax (*Linum usitatissimum* L., 2n = 2x = 30) is a dual-purpose crop that is grown for its stem fiber (for making linen) and oilseed (for making linseed oil). Although fiber and oilseed flax belong to the same species, they exhibit morphologically distinct characteristics, including height and fiber composition, among others. Linen flax fiber is an important raw material for manufacture of textile products. Flax fiber is needed in abundance in China, the top linen exporter worldwide, with Heilongjiang contributing 85% of China's total linen production (FAO, 2014^1^). Meanwhile, with regard to linseed flax, China is the third largest source after India and Canada, with the United States coming in fourth. Gansu, Inner Mongolia, and Xinjiang are the three provinces/regions in China where the majority of linseed flax is grown.

Optimal flax cultivars for textile production can yield flax of high quality, high yield, high fiber content, and other desirable characteristics. However, compared to other crops, little systematic in-depth research has been focused on improving flax cultivars for use with traditional breeding methods. Marker-assisted selection (MAS) would enhance the effectiveness of existing breeding methods to improve flax stem fiber, but currently insufficient molecular resources are available for achieving that goal. Indeed, in spite of the economic importance of flax, until now breeding programs utilizing MAS resources have only been used to construct genetic linkage maps for various crops such as rice (Orjuela et al., 2010; Chen et al., 2012), wheat (Gardner et al., 2016; Holtz et al., 2016), corn (Foiada et al., 2015; Chen et al., 2016), and soybean (Li et al., 2014, 2016; Qi et al., 2014; Song et al., 2015). Fortunately, MAS resources of other crops can provide a valuable foundation of knowledge for future molecular biological studies of flax.

Until recently, flax improvement has relied mainly on conventional breeding methods based upon limited germplasm resources. However, most important agronomic characteristics, such as stem fiber content, are quantitative innate traits that are regulated by micro-effect polygenes and environmental factors. Because increasing the fiber content is an ultimate goal in flax genetics and breeding research, identification of quantitative trait loci (QTL) would be advantageous for MAS and map-based cloning. Unfortunately, a linkage map with suitable marker density as a prerequisite for QTL detection does not yet exist (Thoday, 1961; Paterson et al., 1998). Indeed, only four limited flax linkage maps and one integrated low-density map have been published (Spielmeyer et al., 1998; Oh et al., 2000; Cloutier et al., 2011, 2012; Yi et al., 2017). Moreover, integration of these maps to generate a draft flax genome would require additional information before it could be used to effectively support MAS-based breeding. Therefore, the goal of this study was to construct a suitably dense flax genetic linkage map composed of previously published flax genome assemblies (Wang Z. et al., 2012) in combination with mapped flax fiber trait QTLs discovered in this study. These genetic resources should lay a foundation for further mining of flax genes important for optimal fiber generation for use in MAS-based breeding of flax.

## Results and analysis

### Analysis of SLAF sequencing data and genotyping

**Figure 1:**
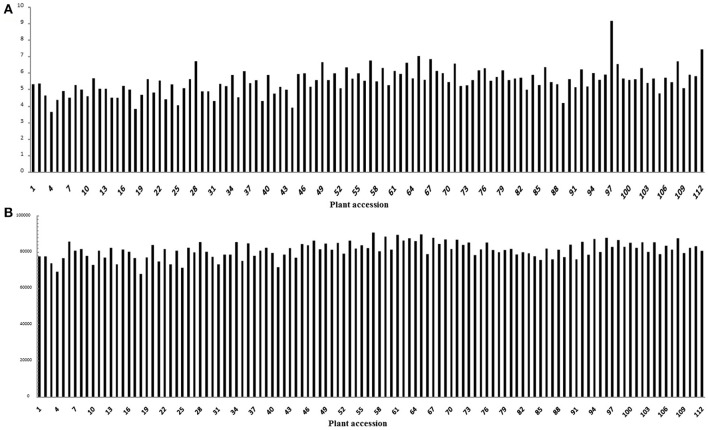
Coverage and number of markers for each of the F2 individuals. The x-axes in both **(A,B)** indicate each of the F_2_ individuals; the y-axes indicate the marker coverage **(A)** and the number of markers developed for each F_2_ plant **(B)**.

A total of 61.64 Gb of raw sequence data was generated using Illumina sequencing after SLAF library construction, which produced 253.71 Mb of paired-end reads, each 101 bp in length. The GC (guanine-cytosine) content was 39.18% and the Q30 ratio (quality scores of at least 30, indicating a 1% chance of error) was 86.64%. For the paternal inbred line (“DIANA”), 9,868,940 reads and 99,043 SLAFs were generated, with an average coverage of 99.64-fold for each SLAF. For the maternal line (“NY17”), the number of reads producing 97,769 SLAFs was 10,899,810 and the average coverage for each SLAF marker was 111.49-fold (Table 1). For the analysis of the F_2_ mapping population, 1,061,346–3,035,345 reads were generated for the development of 67,607–90,504 SLAF markers for each plant, with marker coverage ranging from 3.64- to 9.16-fold for an average coverage of 5.48-fold (Figure 1A). The average count of SLAFs per individual plant was 80,998 (Figure 1B).

**Table 1:** Statistical tables of sequencing data.

**Sample**	**Total reads**	**Q30 percentage(%)**	**GC percentage(%)**
DIANE	9,868,940	86.49	39.45
NY17	10,899,810	86.78	39.42
Offspring	2,025,565	86.64	39.18

**Figure 2:**
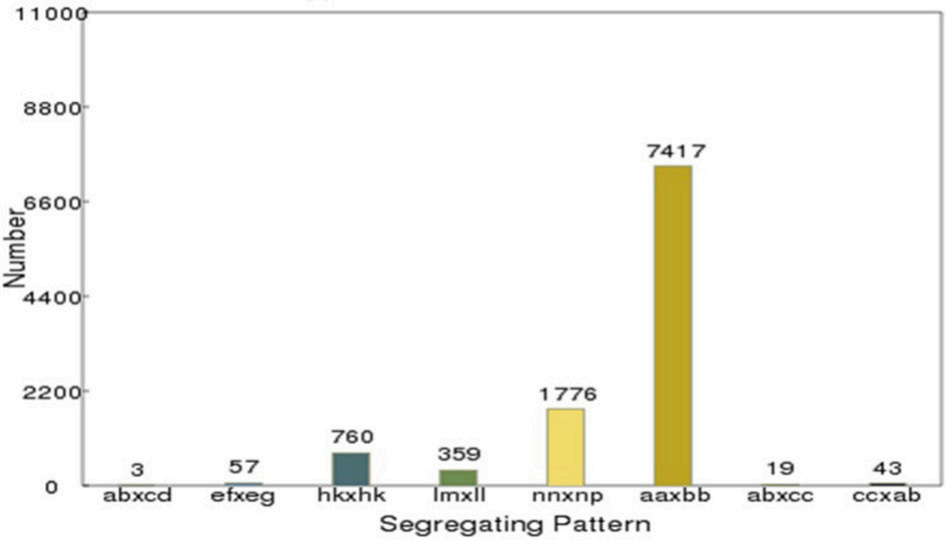
Number of polymorphic SLAF markers for eight segregation patterns. The x-axis indicates eight segregation patterns of polymorphic SLAF markers; the y-axis indicates the number of markers.

After correcting or discarding low-depth SLAF tags, 192,797 high-quality SLAFs were identified, of which 23,115 were polymorphic, for a polymorphism rate of 11.99% (Table 2). The remaining 169,682 SLAFs were non-polymorphic or repetitive. After the parental lines were assigned different alphabetic letter designations as genotypes to determine segregation patterns, 10,434 polymorphic SLAFs were successfully encoded and grouped into eight segregation patterns (ab × cd, ef × eg, ab × cc, cc × ab, hk × hk, lm × ll, nn × np, and aa × bb) following a genotype encoding rule (Figure 2). Since the two parents (“NY17” and “DIANA”) are homozygous inbred lines with genotypes of aa and bb, respectively, 7,417 markers that fell within the aa × bb segregation pattern were used for performance of linkage analysis.

**Table 2:** Discovery of SLAF markers.

**Type**	**Polymorphic SLAF**	**Non-polymorphic SLAF**	**Repetitive SLAF**	**Total SLAF**
Number	23,115	169,340	342	192,797
Percentage (%)	11.99	87.83	0.18	100.00

### Basic characteristics of the genetic map

**Figure 3:**
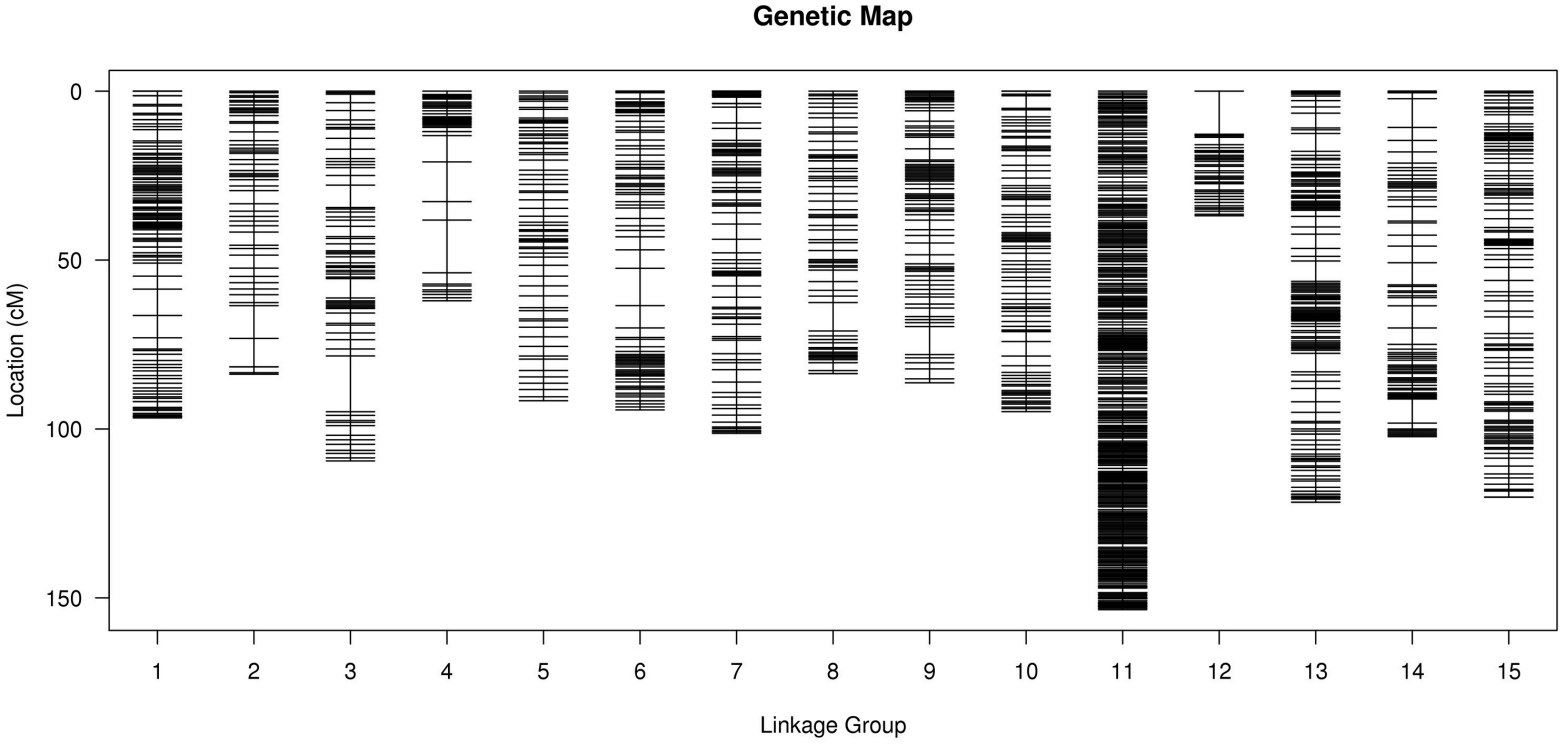
Distribution of SLAF markers on the 15 linkage groups of flax. A black bar indicates a SLAF marker. The x-axis represents the linkage group number and the y-axis indicates the genetic distance (cM) within each linkage group.

After removing incomplete and significant segregation distortion markers, 7,417 SLAFs were retained for the final genetic linkage map construction. The average integrity of the mapped markers was 100%, predicting potential generation of a relatively high quality genetic map. The mLOD values were calculated between pairs of tags following a standard procedure as previously described (Vision et al., 2000). After removal of each tag with a LOD score < 10 when paired with all other tags, 2,339 markers were localized to the map for an overall genome coverage percentage estimate of 31.54% (Table S1). HighMap software (Liu et al., 2014) was used to arrange markers into linkage groups and genetic distances were estimated between adjacent markers. Subsequently, a genetic linkage map of overall length 1,483.25 cM was obtained (Figure 3), with an average distance between adjacent markers of 0.63 cM. The basic characteristics of all linkage groups (LGs) obtained are shown in Table 3. In the overall linkage map, the largest linkage group (LG15) contained 196 SLAFs, while the smallest linkage group (LG12, 34.05 cM) contained 39 SLAFs. LG11 contained the maximum number of markers (765), while LG12 contained the minimum marker number (39). On average, each LG contained 156 SLAF markers. The genetic length of the 15 linkage groups ranged from 34.05 cM (LG12) to 157.28 cM (LG15), with an average interval between adjacent markers ranging from 0.20 cM (LG11) to 1.42 cM (LG2). The “Max Gap,” which reflects the largest degree of linkage between markers, was 16.52 cM and was located in LG3. A total of 5,106 SNP loci were identified among the 2,339 mapped SLAF markers (Table 4) with Tri/Trv values ranging from 1.40 to 2.67. LG11 contained the highest number of SNP loci (1,737), while LG12 contained the lowest number of SNPs (66).

**Table 3:** Basic characteristics of the 15 linkage groups of flax.

**Linkage group**	**Total marker**	**Total distance(cM)**	**Average distance(cM)**	**Max gaps**
LG1	191	96.73	0.51	7.79
LG2	59	83.79	1.42	9.69
LG3	82	109.46	1.33	16.52
LG4	62	65.06	1.05	15.60
LG5	90	91.64	1.02	3.49
LG6	106	94.37	0.89	11.05
LG7	110	101.30	0.92	4.68
LG8	90	83.60	0.93	8.41
LG9	92	86.34	0.94	8.28
LG10	100	94.88	0.95	4.30
LG11	765	153.49	0.20	1.43
LG12	39	34.05	0.87	2.59
LG13	248	128.98	0.52	6.03
LG14	109	102.28	0.94	8.51
LG15	196	157.28	0.80	7.80
Total	2,339	1, 483.25	0.63	16.52

**Table 4:** Distribution of SNP loci on the 15 linkage groups of flax.

**LGs**	**SNP number**	**Tri**	**Trv**	**Tri/Trv**
LG1	486	296	190	1.56
LG2	117	78	39	2.00
LG3	167	99	68	1.46
LG4	119	79	40	1.98
LG5	140	95	45	2.11
LG6	208	123	85	1.45
LG7	240	140	100	1.40
LG8	176	111	65	1.71
LG9	166	102	64	1.59
LG10	193	126	67	1.88
LG11	1737	1079	658	1.64
LG12	66	48	18	2.67
LG13	584	396	188	2.11
LG14	258	167	91	1.84
LG15	449	292	157	1.86
Total	5,106	3,231	1,875	1.72

“*Tri” and “Trv” indicate the transition and transversion numbers, respectively*.

### Quality evaluation of the genetic map

**Figure 4:**
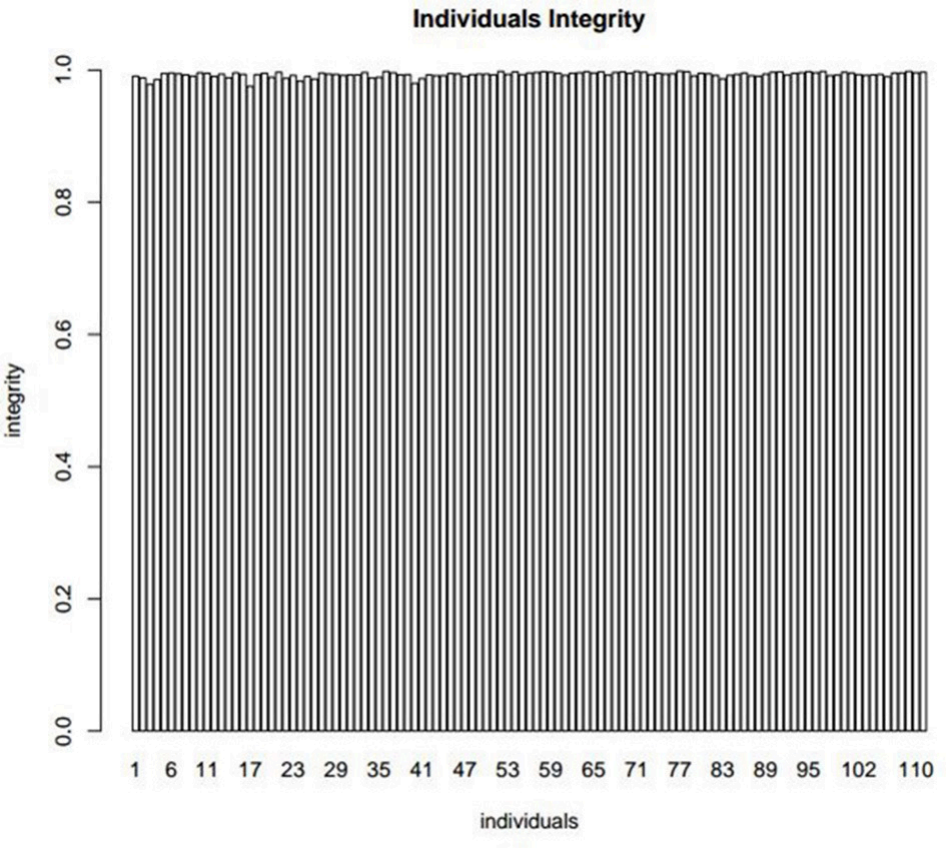
The integrity distribution map of all individuals. The x-axis represents the 112 individuals and y-axis represents the complete degree of mapped markers.

The quality of the flax genetic map was evaluated by analyzing the integrity of mapped markers. The average integrity of each individual marker was 99.33% (Figure 4), the average depth of offspring was 7.72, and the parental depth was five times greater than the depth of offspring (Table 5), all of which suggest genotyping accuracy (Table S2).

**Table 5:** Statistics of the mapped marker depth.

**Samples**	**Marker numbers**	**Total depth**	**Average depth**
Ningya No.17	2,339	84,733	36.23
DIANA	2,339	80,269	34.32
Average of Offspring	2,108	16,270	7.72

Haplotype maps, which reflect chromosomal-exchange events among genomes within a population, were developed for the 112 offspring and parental controls using the 2,339 SLAF markers (Presentation S1). Dual-exchange sites might result from two scenarios: (1) a recombinant hotspot region within the genome and (2) genotyping error caused by sequencing within a linkage group. Consequently, a higher proportion of dual-exchange events correlated with greater success of genotyping and more effective ordering of markers. In this study, the percentage of dual-exchanges ranged from 0.01% (LG1) to 0.25% (LG12), while the percentage of missing data for each LG ranged from 0.01% (LG15) to 2.92% (LG9) (Table 6). Ultimately, most recombination blocks were clearly defined and the LGs were uniformly distributed, suggesting that genetic mapping was of high quality.

**Table 6:** Double exchange and missing ratio of the mapped markers.

**Linkage group ID**	**Singleton percent (%)**	**Missing percent (%)**
LG1	0.01	0.07
LG2	0.11	0.24
LG3	0.09	2.44
LG4	0.16	0.16
LG5	0.16	0.21
LG6	0.13	0.16
LG7	0.02	2.69
LG8	0.06	2.57
LG9	0.04	2.92
LG10	0.04	1.80
LG11	0.09	0.04
LG12	0.25	0.92
LG13	0.03	0.04
LG14	0.05	2.06
LG15	0.03	0.01

A genetic map essentially reflects multipoint recombination analysis, with closer distances between adjacent markers reflecting smaller observed recombination rates. To analyze recombination relationships between markers, we determined the potential layout of mapped markers. The quality of the genetic map was also evaluated using heat maps which directly depicted recombination relationships among markers for all fifteen linkage groups (Presentation S2). Each cell of the heat map represents a recombination rate between two adjacent markers whereby the rate level was depicted using different colors ranging from yellow to purple (yellow indicating a lower recombination rate; purple indicating a higher rate). In this way, heat maps were generated for each LG using recombination scores for pair-wise comparative analyses of the 2,339 markers (Presentation S2). The resulting heat maps indicate that SLAF markers in most LGs were well ordered.

### QTL mapping for related traits

**Figure 5:**
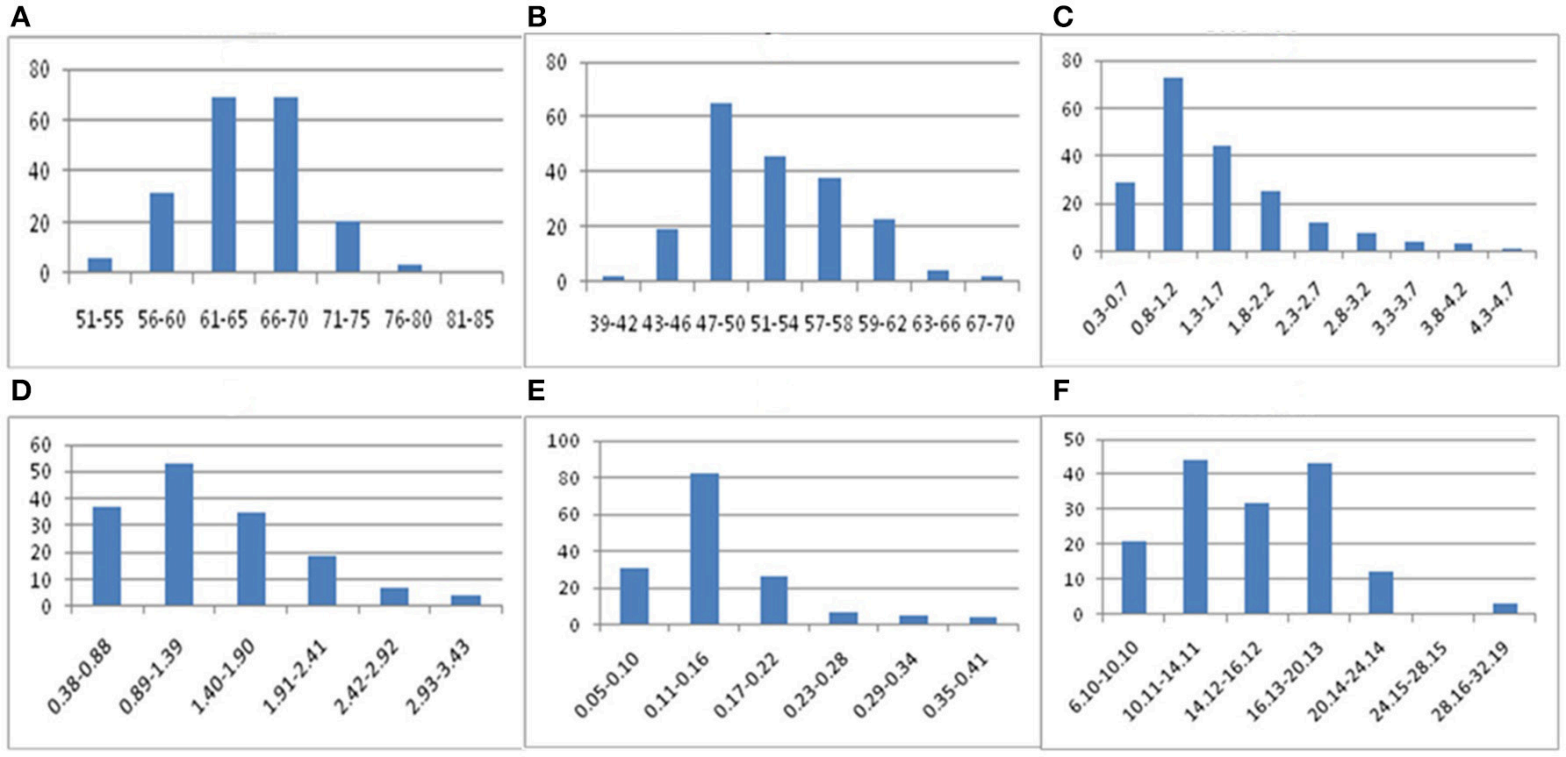
Frequency distribution of flax fiber related traits. (**A**: plant height; **B**: stem length; **C**: seed yield; **D**: stem yield; **E**: fiber yield; **F**: fiber content).

Phenotypic data for F_2_ families are presented in Table 7 and the frequency distribution of all measured traits is shown in Figure 5. Plant height, stem length, and stem yield all strictly followed normal distributions, with kurtosis and skewing values close to zero. Meanwhile, the frequency distributions of seed yield and fiber content exhibited approximately normal distributions, but with higher kurtoses (although only the left peaks were characterized). The results indicate that quantitative traits are controlled by multiple genes, with overall trait values of offspring biased approaching value of the pure-bred parents. These results therefore suggest a heterosis degradative phenomenon may be operating for some traits in flax.

**Table 7:** General statistics of offspring.

**Traits**	**Mean**	**Standard error**	**Range**	**Kurtosis**	**Skewness**
Plant height/cm	65.38	0.52	54.00 ~ 84.00	0.37	0.33
Stem length/cm	52.09	0.51	40.00 ~ 68.00	0.69	0.61
Seed yield/g	1.62	0.08	0.30 ~ 4.70	1.50	1.15
Stem yield/g	1.46	0.06	0.55 ~ 3.40	0.28	0.77
Fiber yield/g	0.16	0.01	0.06 ~ 0.41	2.65	1.52
Fiber content/%	14.86	0.44	6.15 ~ 32.19	1.64	0.83

Based on the assembly information of flax genome, which chromosome-scale pseudomolecules were published (You et al., 2018), it is now possible to realize the positions of QTLs for various traits once all traits are mapped to the scaffold fragments over the entire genome. A total of 12 QTLs were detected within the linkage groups (Table 8), including a QTL detected for plant height on chrLG1 linked with scaffold156, with a LOD score of 3.85 that accounted for 18.77% of the total phenotypic variance for that trait., therefore, the candidate gene of the target trait may actually reside nearby. For stem length, a QTL with LOD score of 3.17 was detected within chrLG8 linked with scaffold2786 that accounted for 11.17% of the total phenotypic variance. Meanwhile, three QTLs were detected for seed yield on chrLG10 and chrLG12 (linked with the scaffold319, scaffold117, and scaffold27), with LOD scores of 2.73, 2.13, and 2.11 that accounted for 10.11–19.33% of the total phenotypic variance. In addition, three QTLs were detected for stem yield on chrLG5 and chrLG15 (linked with the scaffold33 and scaffold355), with LOD scores of 2.97, 2.62, and 2.70 that accounted for 10.91–15.81% of the total phenotypic variance. Two QTLs were detected for fiber yield on chrLG1 and chrLG11 (linked with the scaffold156 and scaffold127), with LOD scores of 3.31 and 3.68, respectively, accounting for 19.09–25.98% of the total phenotypic variance. Finally, two QTLs were detected for fiber content on chrLG5 and chrLG11 (linked with the scaffold680 and scaffold376), with LOD scores of 3.51 and 3.91 that accounted for 15.14 and 13.27% of the total phenotypic variance, respectively.

**Table 8:** QTL analysis of fiber related traits in F2 populations.

	**chrLG**	**Pos.IM (cM)**	**Closest marker**	**LOD**	**PVE**.	**Scaffold**
Plant height	1	87.85	Marker4371	3.85	18.77	Scaffold156
Stem length	8	2.23	Marker747228	3.17	11.17	Scaffold2786
Seed yield	10	51.34	Marker799956	2.73	10.11	Scaffold319
	12	7.67	Marker770415	2.13	19.33	Scaffold117
	12	17.31	Marker1073071	2.11	18.63	Scaffold27
Stem yield	5	75.55	Marker326151	2.97	15.26	Scaffold33
	15	12.17	Marker2368217	2.62	15.81	Scaffold355
	15	48.96	Marker614116	2.70	10.91	Scaffold355
Fiber yield	1	84.19	Marker2603286	3.31	19.09	Scaffold156
	11	71.33	Marker1722134	3.68	25.98	Scaffold127
Fiber content	5	72.71	Marker1051901	3.51	15.14	Scaffold680
	11	76.74	Marker1561746	3.91	13.27	Scaffold376

*Pos.IM (cM) indicate the peak of genetic position of the QTL in the map; LOD, likelihood-ratio statistic, the test is based on the likelihood ratio, which expresses how many times more likely the QTL is reality model than the absence. PVE, the percentage of phenotypic variance explained by each QTL based on the population variance found within the segregation population*.

Interestingly, main effect QTLs for plant height and fiber yield were both detected to the same extent on chrLG1 which spanned the genetic distance from 87.85 to 84.19 cM and contained the same scaffold156. A similar result was observed for stem length, although the different genetic distance spanned, whereby the main effect QTLs were detected to the same scaffold within chrLG5. Therefore, a bold idea emerged that a pleiotropic gene or neighboring genes involved in plant height and/or fiber yield is likely to exist within shared DNA fragments. All of these anchored QTLs should serve as a foundation for later accurate identification of related genes.

Previously, flax QTL mapping based on the genetic linkage map alone could only successfully map a few markers or segments linked to a particular target trait gene, but could not localize target trait genes to physical chromosomal regions. In this study using genomic assembly information in addition to QTL mapping, target trait QTLs could be identified on chromosomes and could be localized to smaller chromosomal segments. However, because each segment may contain several or even dozens of genes, further localization and identification of target genes will still require great effort. Nevertheless, the results presented here provide a foundation for further flax gene mining and characterization.

## Discussion

Large-scale genotyping methodologies play an important role in genetic association studies. One such technology, SLAF-seq, has been applied in many plant studies and has produced remarkable results. Due to its relatively higher density, excellent consistency, effectiveness, and lower cost when compared with traditional methods, this method has become a popular genotyping method. Indeed, in our previous study SLAF-seq technology was used to develop numerous novel SSRs in flax (Wu et al., 2017). Subsequently, in this work sequencing results provided a large quantity of SLAF markers to further drive flax genomic research. Moreover, the SLAFs developed here may also be valuable in studies of other flax cultivars and offspring for identification of germplasm or hybrids and for analysis of genetic diversity among cultivars.

SLAF-seq has been widely successful due to several distinguishing characteristics: (i) deep sequencing ensures genotyping accuracy; (ii) reduced representation strategy reduces sequencing costs; (iii) a pre-designed reduced representation scheme optimizes marker efficiency; and (iv) a double barcode system facilitates sequencing of large populations (Sun et al., 2013). Consequently, this technology has been used in numerous genetic linkage map construction studies (Zhang et al., 2013, 2015; Li et al., 2014; Liu et al., 2014, 2016; Wei et al., 2014; Guo et al., 2015; Jiang et al., 2015; Ma et al., 2015; Xu et al., 2015; Zhu et al., 2015, 2016; Yi et al., 2017). In the present study, the total length of the new genetic linkage map, which was constructed using 2,339 SLAF markers, was 1,483.25 cM and spanned 15 LGs with an average distance between adjacent markers of 0.63 cM. To our knowledge, this map is the highest density genetic map of flax currently available and is of high quality.

The general steps of genome assembly mainly include: determination, arrangement, and directional orientation of scaffolds/contigs onto chromosomes (Burton et al., 2013). Although sequencing completed to date toward obtaining the entire flax genome sequence has produced high-quality genomic information, assembly has been difficult due to the high complexity of the genome. However, much work is still needed before the entire flax genome can be assembled, due to genomic complexity and a large number of repeated regions within the genome (Wang Z. et al., 2012). As the carrier of flax genetic information, the genome is the basis for research on genetic mechanisms of flax. Fortunately, chromosome-scale pseudomolecules were refined by optical, physical, and genetic maps in flax (You et al., 2018), this information provides a genome-wide scaffold-based chromosome assembly to guide future physically mapped of target genes. We employed the genome assembly information, with the high-density map of the flax genome constructed here, 12 QTLs were detected and linked with the chromosome scaffolds. Although the order is different in chrLGs and chromosome-scale pseudomolecules, it still in conjunction with sequence information to generate 15 chromosomes. Notably, this method has generated ideas and strategies with wider applicability for use in whole genome assembly of related species.

Interestingly, characteristic fiber-related traits were observed to segregate in flax F_2_ offspring. Since “DIANE” is a cultivar of fiber flax and “NY17” is a cultivar of oilseed flax, crossing these cultivars may therefore result in generation of new varieties with high fiber content and would provide information regarding inheritance of fiber-based traits. Moreover, because plant phenotypic traits of height, stem length, stem yield, seed yield, fiber yield, and fiber content exhibit characteristics of quantitative traits, the mapped QTLs of these fiber-related traits to physical locations on chromosome scaffolds. Moreover, the results have helped us make provisional inferences of pleiotropic genes or neighboring genes that influence plant height and fiber yield to explain observed morphological correlations. Selection of new cultivars with high fiber content should thus allow for economically feasible flax production. In addition, our findings suggest that reduced representation genome sequencing (RRGS) might be effectively utilized for QTL mapping of fiber related traits in flax. Using this strategy, the major QTL identified here would serve as a promising starting point for the study of genetic mechanisms influencing fiber yield in flax. Collectively, the results of this study should greatly facilitate subsequent fine mapping of target genes and MAS-based breeding toward the goal of determining the whole flax genome.

## Experimental procedures

### Plant materials

The F_2_ mapping population consisted of 112 individuals from the intraspecific crossing of *L. usitatissimum* L. “DIANE” (male parent, fiber flax) and “NY17” (female parent, oilseed flax). All individual progeny and parent cultivars were grown at the Institute of Industrial Crops of Heilongjiang Academy of Agricultural Sciences, Harbin, China. “DIANE” is a typical fiber flax variety that attains a maximum plant height of 98.2 ~ 110.5 cm and exhibits a thousand seed weight (TSW) of 4.3 ~ 5.0 g. An important trait of “DIANE” is its resistance to saline-alkaline soil and a high fiber content yield. “NY17” is an oil flax variety that has been used for dual production of oil and fiber, with a maximum plant height of 52.9 ~ 62.6 cm and a TSW of 7.3 ~ 8.4 g. An important trait of “NY17” is its high oil content ratio. Field management adhered to essentially standard breeding practices and all flax materials were harvested on time when seeds were mature. All traits were investigated or measured as described at http://www.cgris.net/.

### Phenotypic characterization

In this study, phenotypic descriptions of flax agronomic traits were performed as follows:

Plant height: the height from the junction of cotyledons with the stem to the top of the plant in centimeters (cm); Stem length: the distance between the junction of cotyledons and stem to the base of the first branch (cm); Seed yield: seed weight per plant, with grams (g) as the unit; Stem yield: after removal of soil, leaves, and capsules after plants were threshed, the original stem weight of each plant is called the stem yield (g); Fiber yield: the yield of the stem fiber stripped from the dried stem (g). After measuring plant height, stem length, threshing yield, and seed yield, stem yield was determined after the retting process, fiber yield was determined after removing the hards, and fiber content was calculated as the proportion of the total fiber yield contributed by post-retting stem yield.

### DNA extraction

Young healthy leaves from both parents and 112 F_2_ individuals were collected, frozen in liquid nitrogen, then used for DNA extraction. Total genomic DNA was prepared from each plant according to the manufacturer's instructions using a Qiagen DNeasy 96 Plant Kit (Qiagen, CA, USA). DNA concentration and quality were estimated using an ND-1000 spectrophotometer (NanoDrop, DE, USA). Genomic DNA was visualized by electrophoresis on 0.8% agarose gels and was quantified using a NanoDrop 2000 Spectrophotometer (Thermo Scientific, MA, USA). DNA concentration was measured and adjusted to the same level for all samples for use in construction of the SLAF library.

### SLAF library construction and high-throughput sequencing

**Figure 6:**
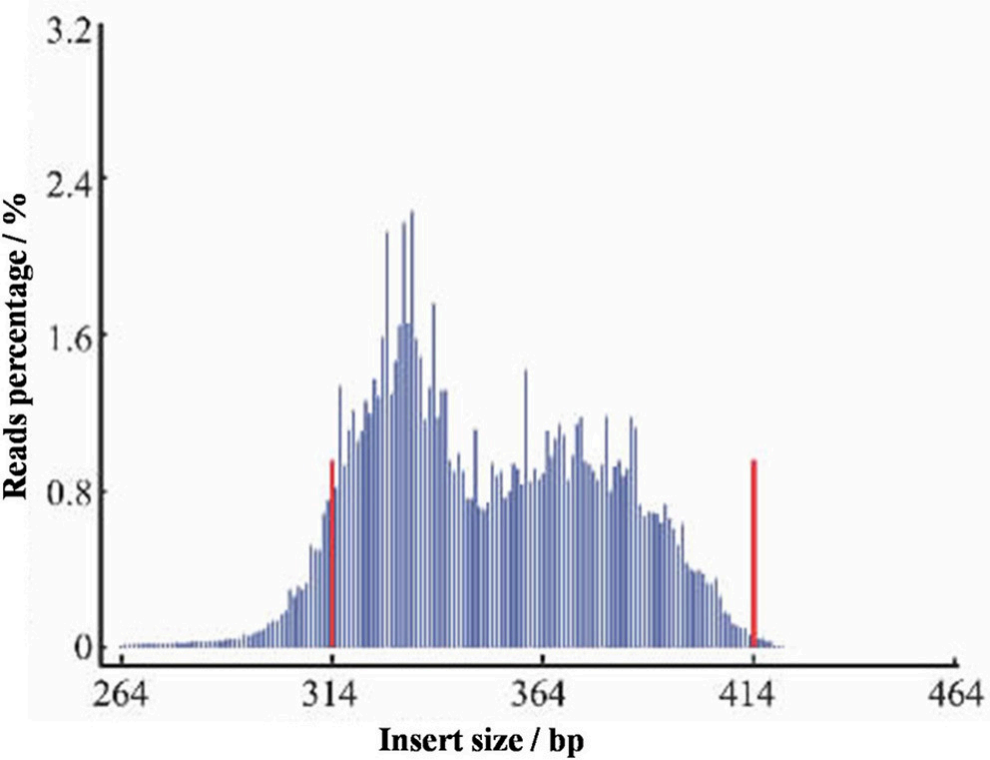
Distribution map of the insert fragment. The x-axis represents the length of the inserted fragment of reads, and y-axis represents the number of reads.

Here, the *L. usitatissimum* L. assembled genome size was only 316 Mb with a GC content of 40%. A reference genome of estimated size of 373 Mb was downloaded from GenBank (NCBI GenBank under GenomeProject ID #68161 and Sequence Read Archive accession SRA038451, https://www.ncbi.nlm.nih.gov/sra/?term=SRA038451). Optimal enzymes used to digest DNA were chosen according to the reference genome sequence in combination with the GC content of flax. The SLAF library was constructed based on a pilot SLAF experiment. After samples were purified, genomic DNA was digested using restriction enzymes *Rsa*I and *Hae*III. Next, using a dual-indexing strategy (The Arabidopsis Genome Initiative, 2000) dATP was used to add a single nucleotide (A) overhang to the digested fragments at 37°C. Next, T4 DNA ligase was used to ligate duplex tag-labeled sequencing adapters (polyacrylamide gel electrophoresis (PAGE)-purified; Life Technologies, USA) to the A-tailed fragments. DNA fragments (SLAFs) of 314–414 bp (with indexes and adaptors) were excised and diluted for paired-end sequencing on an Illumina HiSeq 2000 sequencing platform (Illumina, Inc; San Diego, CA, USA) conducted by Biomarker Technologies Corporation in Beijing (http://www.biomarker.com.cn/english/) (Figure 6). Real-time monitoring was performed for each cycle during sequencing and ultimately the ratio of high quality raw reads with quality scores greater than Q30 (quality score >30 indicates a 1% chance of error and thus 99% confidence) were used for subsequent analyses. Guanine-cytosine (GC) content was calculated for quality control. Gel-purified products were then diluted and high-throughput paired-end sequencing (101 bp from both ends) was performed using an Illumina HiSeq 2500 System (Illumina, Inc., San Diego, CA, USA) according to the manufacturer's instructions.

### SLAF-seq data grouping and genotype definition

SLAF marker identification and genotyping were performed using procedures described by Sun et al. (2013). Low-quality reads (quality score < 30) were deleted and the remaining raw reads were assigned to 112 individual plant samples according to duplex barcodes of sequences. After trimming the barcodes and the terminal 5-bp positions from each high-quality read, clean reads were clustered together according to their sequence identities. Sequences mapping to the same locus with over 90% identity were assigned to a single SLAF locus (Zhang et al., 2015).

SLAFs with 2, 3, or 4 tags were identified as polymorphic SLAFs and considered to be potential markers. Polymorphic markers were classified into eight segregation patterns (ab × cd, ef × eg, hk × hk, lm × ll, nn × np, aa × bb, ab × cc, and cc × ab). The genetic map was constructed using the F_2_ population from a cross between two heterozygous parents after the markers from the segregation pattern of aa × bb were filtered out. Average sequence depths of SLAF markers were >20-fold for parents and >14-fold for progeny. All progeny contained more than 80% of the SLAF markers of the parents, i.e., 80% integrity of SLAF markers were observed in individual plants.

### Genetic map construction and quality evaluation

After removing incomplete and significant segregation distortion markers, 7,417 SLAFs were retained for genetic map construction using a logarithm of odds (LOD) threshold ≥3.0 and a maximum recombination fraction of 0.4, as described by van Ooijen (2011). A HighMap strategy was employed to order the SLAF markers according to the protocol detailed by Liu et al. (2014). Genotyping errors were corrected by the SMOOTH algorithm (van Os et al., 2005). Missing genotypes were imputed using a k-nearest neighbor algorithm (Huang et al., 2011). Map distances in cM were calculated using the Kosambi mapping function (Kosambi, 1944).

Genetic map quality was evaluated using three criteria. The integrity of mapped markers was analyzed in order to correct marker ordering to produce better results. Haplotype maps and heat maps were constructed that directly reflected recombination relationships for determination of the maximum possible double exchange sites among markers within the fifteen linkage groups. Haplotype maps were used to count double exchange and deletion events for all linkage groups such that positions indicated by sites marked by color changes were where reorganization events occurred. Each cell represents a recombination rate between two adjacent markers for each heat map, with recombination rates depicted using different colors ranging from yellow to purple (yellow indicating a lower recombination rate; purple indicating a higher rate). Heat maps also detected potential marker ordering conflicts.

### QTL analysis

QTL analysis was carried out using an R/QTL procedure employing an interval mapping method as previously described (Peichel et al., 2001). Composite interval mapping (CIM) was adopted using a walking speed of 1 cM (Wang W. X. et al., 2012). The significance of each QTL interval was tested by determining a statistical likelihood-ratio (logarithm of the odds or LOD score). The threshold of significance of the LOD score (*P* = 0.05) was determined using a permutation test (PT) of 1,000 permutations. First, the threshold value was set using the 1,000-permutation test to determine the LOD significance threshold value corresponding to a 0.99 confidence level. If there was no mapping interval, 2-LOD support intervals were constructed using 0.95 confidence intervals (van Ooijen, 1992). If the results of the PT test were not considered, the threshold value was reduced to 3.0 manually. If no interval was observed, then the 3.0 threshold was reduced to 2.0. Calculation of the percentage of phenotypic variance explained (PVE%) by each QTL was obtained from the QTL peak area based on the population variance found within the segregating population.

## Author contributions

JW and QZ conceived and designed the research experiments. JW, GW, HY, and DS implemented the filed experiments. JW, LZ, YY, and TJ performed the phenotyping and genotyping. JW and QZ analyzed the data. JW and TJ designed the overall project. JW wrote the manuscript. JW, ZL, XY, and XW revised the final version of the manuscript. All authors reviewed and approved the final manuscript.

### Conflict of interest statement

The authors declare that the research was conducted in the absence of any commercial or financial relationships that could be construed as a potential conflict of interest.

## Footnotes

^1^Food and Agriculture Organization. http://www.fao.org/home/en/

## Supplementary material

Table S1 Summary of the sequencing data.

Table S2 SLAF marker numbers and depth of the sequencing data.

Presentation S1 Haplotype map of the genetic maps. Each row represents a marker. Markers are ranked in accordance with the map order. Each of the two columns represents an individual plant allelic set, with green and blue shading of columns representing the parental alleles. The white column represents that the source of alleles cannot be confirmed. The gray columns represent the deleted alleles.

Presentation S2 Heat map of the genetic maps. Markers of each row and column are ranked according to the map order; each small square represents the rate of recombination (r) between the two markers.
