# Supplementary figures and images for: QTL Mapping of Fiber-Related Traits Based on a High-Density Genetic Map in Flax (Linum usitatissimum L.)
Source: Front Plant Sci. 2018 Jul 17;9:885. doi: 10.3389/fpls.2018.00885 (PMC6056681; doi:10.3389/fpls.2018.00885)

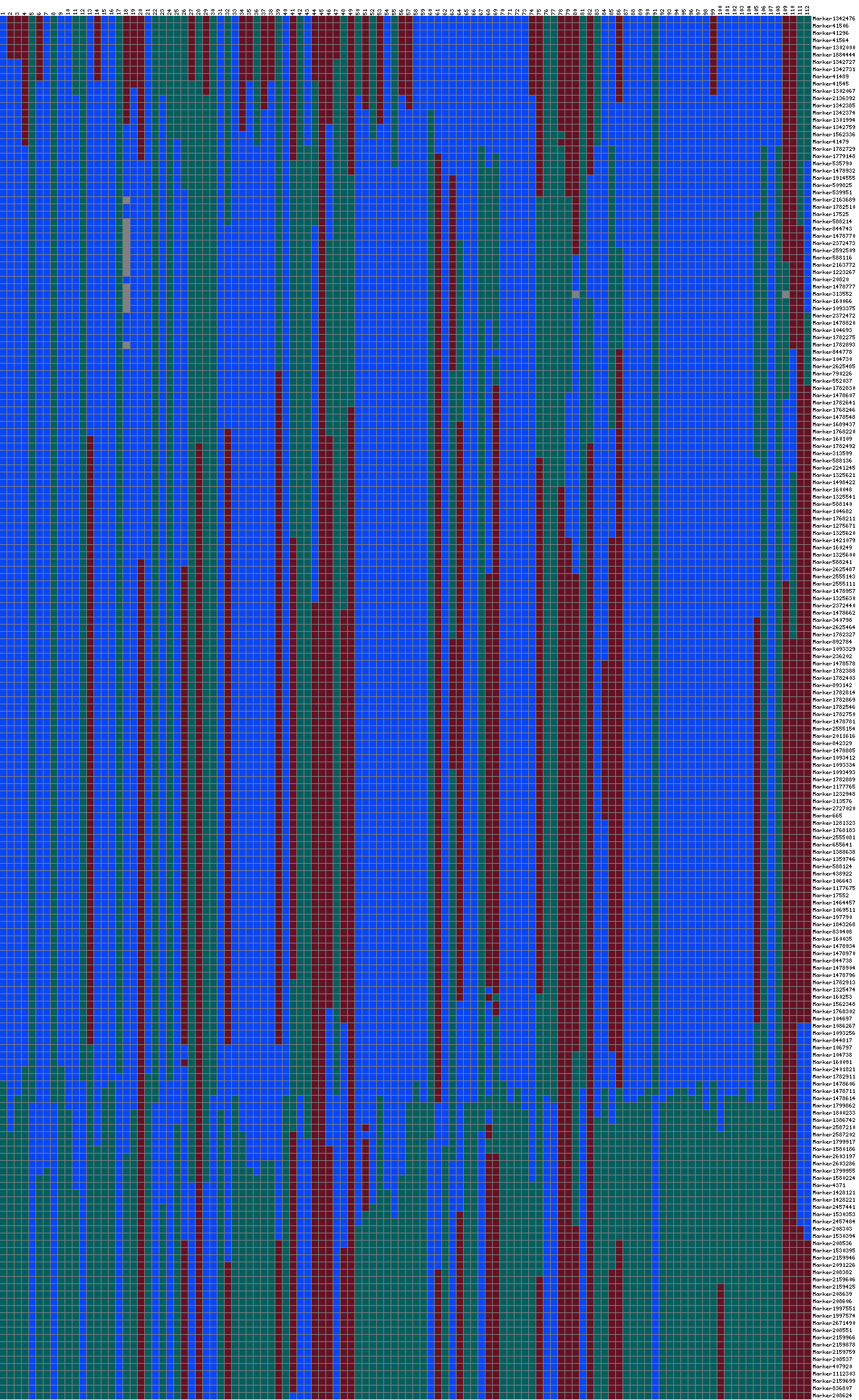

Supplement: Presentation S1 — Haplotype map of the genetic maps. Each row represents a marker. Markers are ranked in accordance with the map order. Each of the two columns represents an individual plant allelic set, with green and blue shading of columns representing the parental alleles. The white column represents that the source of alleles cannot be confirmed. The gray columns represent the deleted alleles. [file Presentation_1.ZIP › supplementary material presentation 1/LG1.haplo.png]

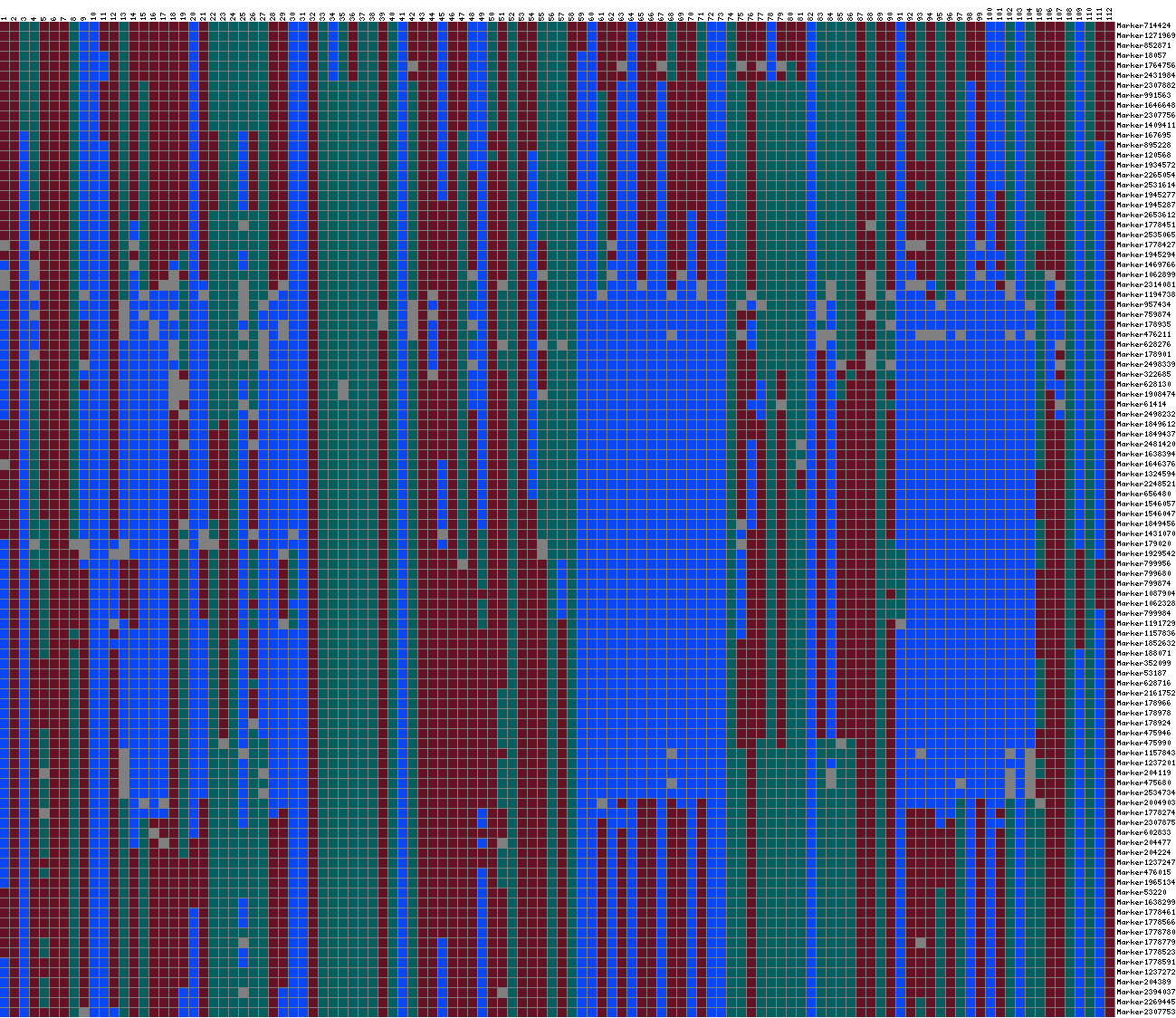

Supplement: Presentation S1 — Haplotype map of the genetic maps. Each row represents a marker. Markers are ranked in accordance with the map order. Each of the two columns represents an individual plant allelic set, with green and blue shading of columns representing the parental alleles. The white column represents that the source of alleles cannot be confirmed. The gray columns represent the deleted alleles. [file Presentation_1.ZIP › supplementary material presentation 1/LG10.haplo.png]

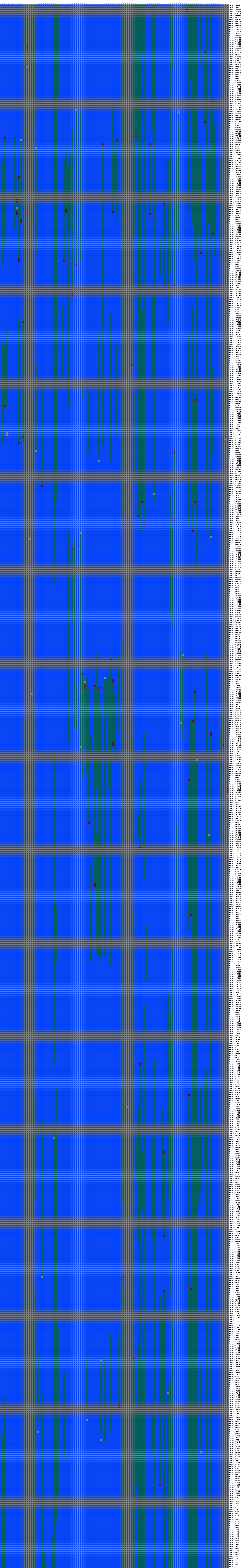

Supplement: Presentation S1 — Haplotype map of the genetic maps. Each row represents a marker. Markers are ranked in accordance with the map order. Each of the two columns represents an individual plant allelic set, with green and blue shading of columns representing the parental alleles. The white column represents that the source of alleles cannot be confirmed. The gray columns represent the deleted alleles. [file Presentation_1.ZIP › supplementary material presentation 1/LG11.haplo.png]

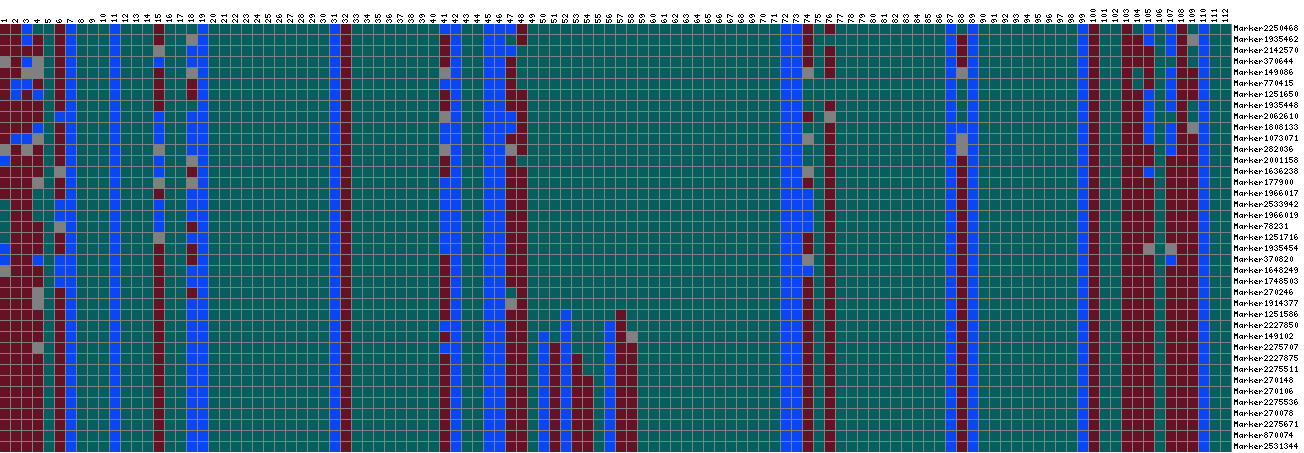

Supplement: Presentation S1 — Haplotype map of the genetic maps. Each row represents a marker. Markers are ranked in accordance with the map order. Each of the two columns represents an individual plant allelic set, with green and blue shading of columns representing the parental alleles. The white column represents that the source of alleles cannot be confirmed. The gray columns represent the deleted alleles. [file Presentation_1.ZIP › supplementary material presentation 1/LG12.haplo.png]

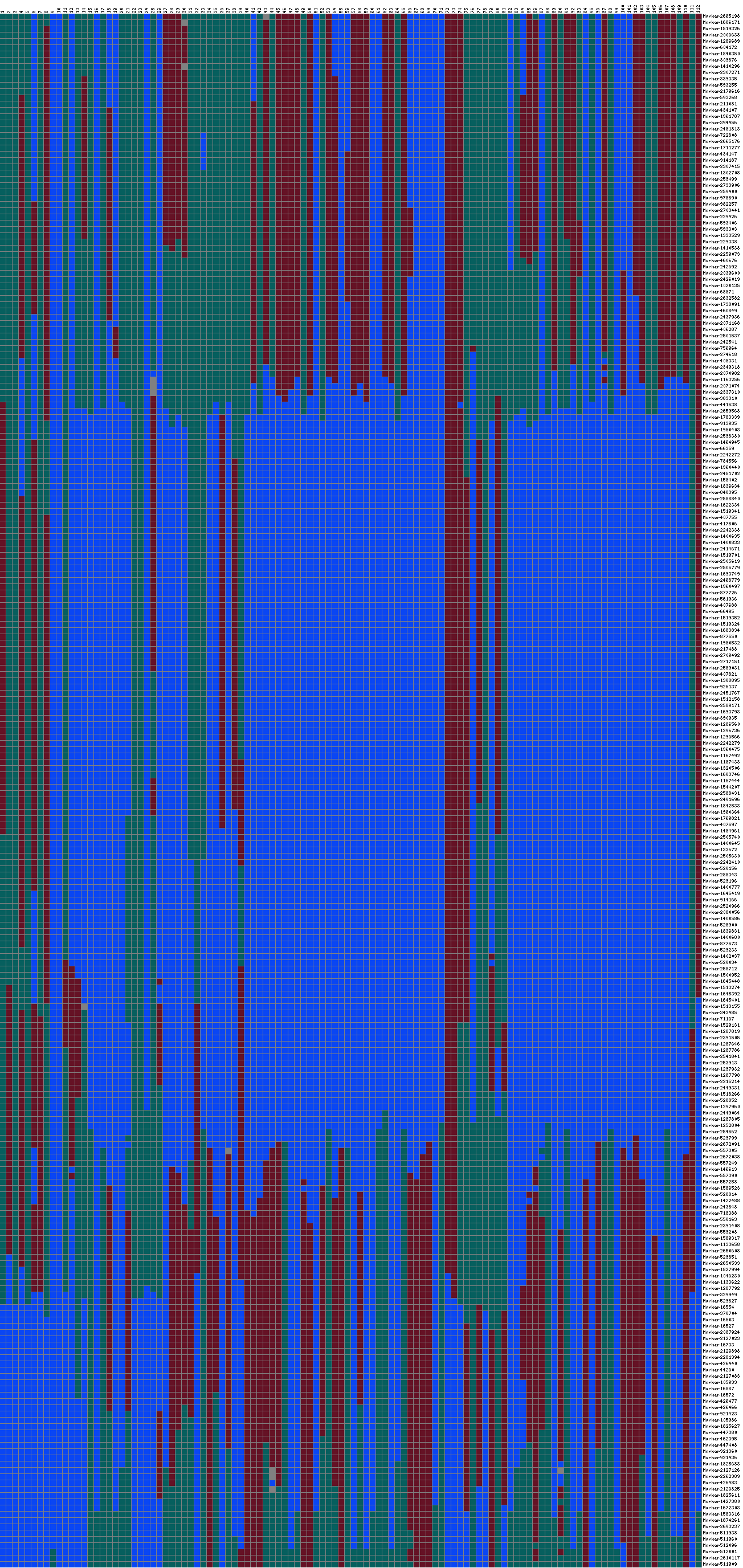

Supplement: Presentation S1 — Haplotype map of the genetic maps. Each row represents a marker. Markers are ranked in accordance with the map order. Each of the two columns represents an individual plant allelic set, with green and blue shading of columns representing the parental alleles. The white column represents that the source of alleles cannot be confirmed. The gray columns represent the deleted alleles. [file Presentation_1.ZIP › supplementary material presentation 1/LG13.haplo.png]

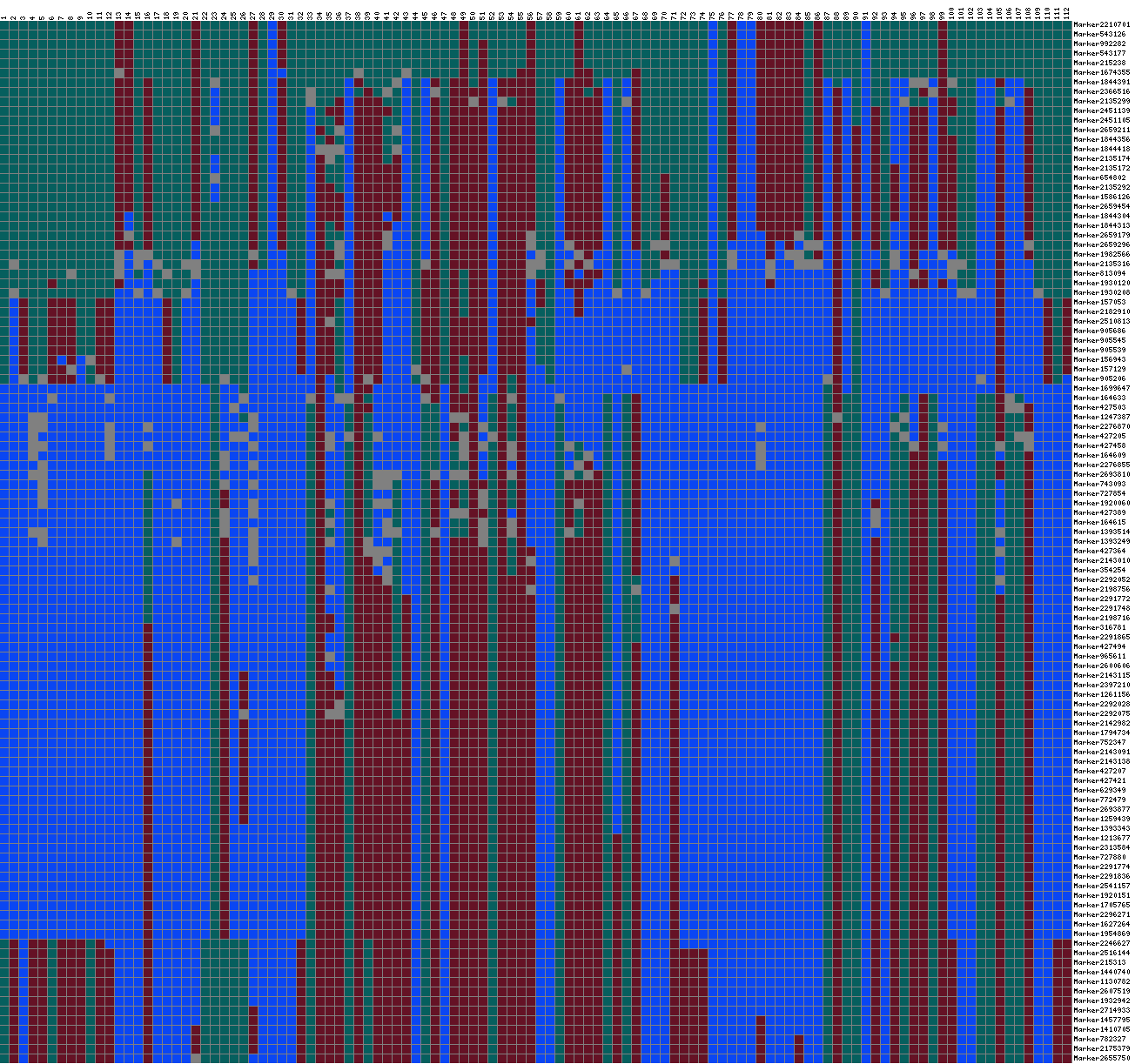

Supplement: Presentation S1 — Haplotype map of the genetic maps. Each row represents a marker. Markers are ranked in accordance with the map order. Each of the two columns represents an individual plant allelic set, with green and blue shading of columns representing the parental alleles. The white column represents that the source of alleles cannot be confirmed. The gray columns represent the deleted alleles. [file Presentation_1.ZIP › supplementary material presentation 1/LG14.haplo.png]

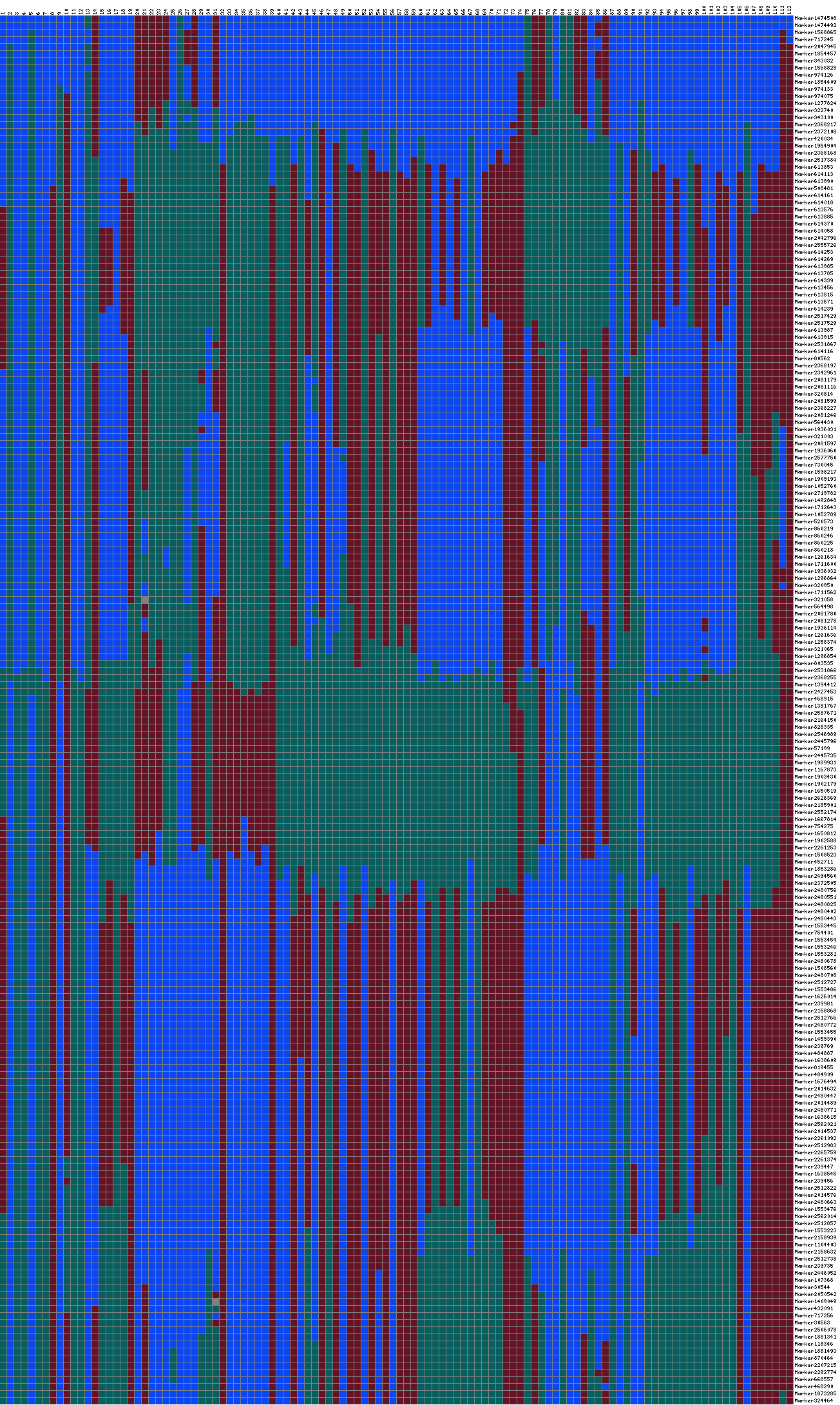

Supplement: Presentation S1 — Haplotype map of the genetic maps. Each row represents a marker. Markers are ranked in accordance with the map order. Each of the two columns represents an individual plant allelic set, with green and blue shading of columns representing the parental alleles. The white column represents that the source of alleles cannot be confirmed. The gray columns represent the deleted alleles. [file Presentation_1.ZIP › supplementary material presentation 1/LG15.haplo.png]

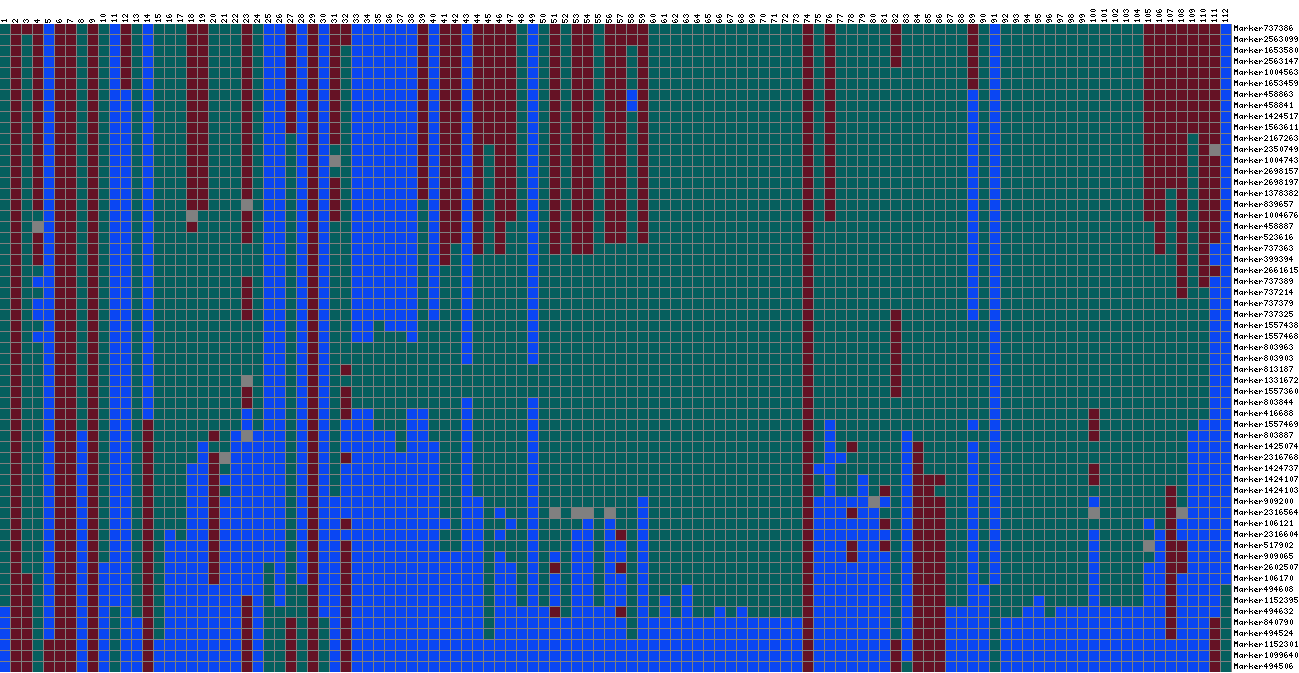

Supplement: Presentation S1 — Haplotype map of the genetic maps. Each row represents a marker. Markers are ranked in accordance with the map order. Each of the two columns represents an individual plant allelic set, with green and blue shading of columns representing the parental alleles. The white column represents that the source of alleles cannot be confirmed. The gray columns represent the deleted alleles. [file Presentation_1.ZIP › supplementary material presentation 1/LG2.haplo.png]

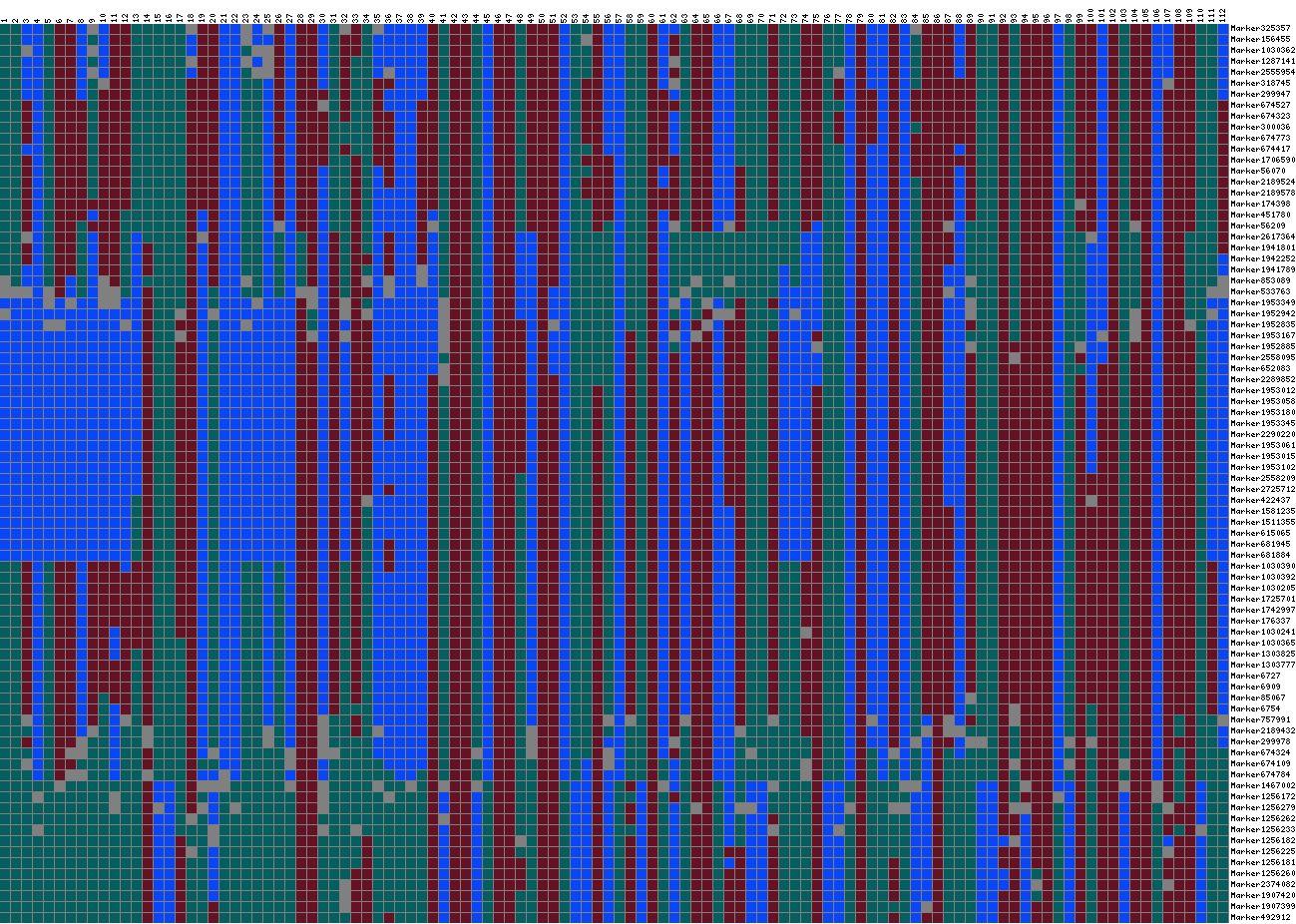

Supplement: Presentation S1 — Haplotype map of the genetic maps. Each row represents a marker. Markers are ranked in accordance with the map order. Each of the two columns represents an individual plant allelic set, with green and blue shading of columns representing the parental alleles. The white column represents that the source of alleles cannot be confirmed. The gray columns represent the deleted alleles. [file Presentation_1.ZIP › supplementary material presentation 1/LG3.haplo.png]

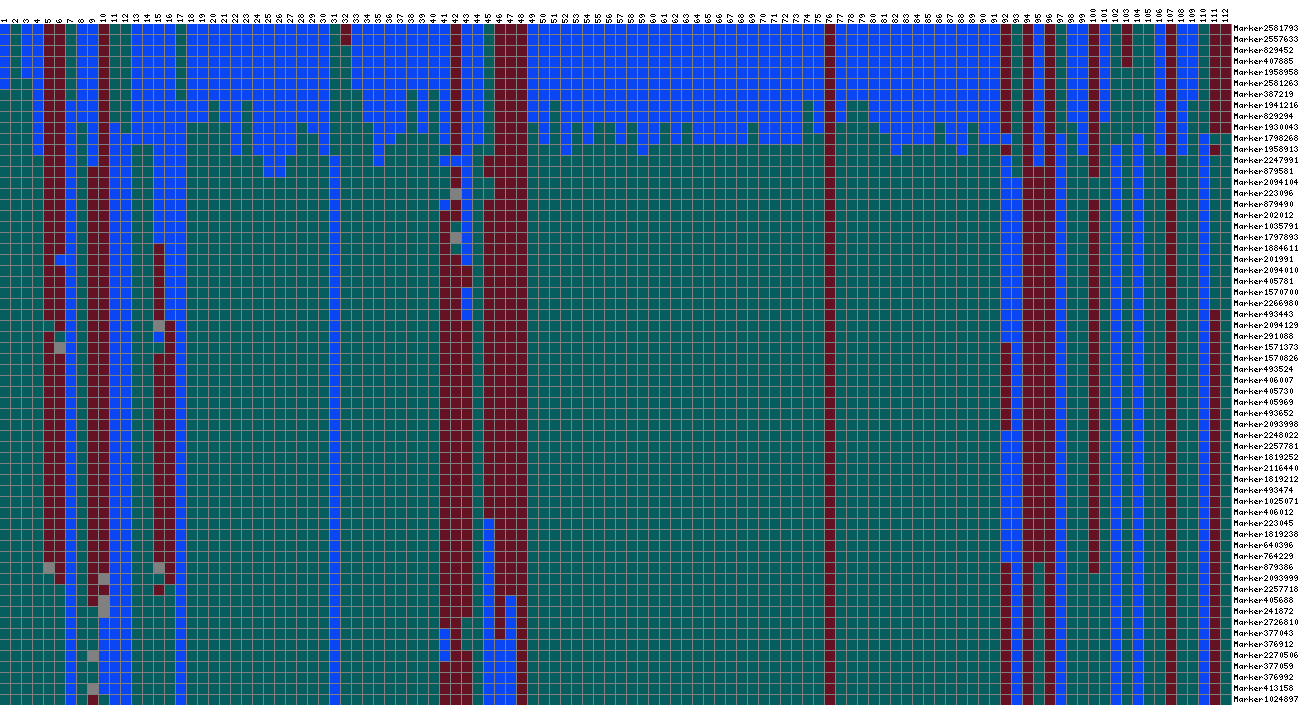

Supplement: Presentation S1 — Haplotype map of the genetic maps. Each row represents a marker. Markers are ranked in accordance with the map order. Each of the two columns represents an individual plant allelic set, with green and blue shading of columns representing the parental alleles. The white column represents that the source of alleles cannot be confirmed. The gray columns represent the deleted alleles. [file Presentation_1.ZIP › supplementary material presentation 1/LG4.haplo.png]

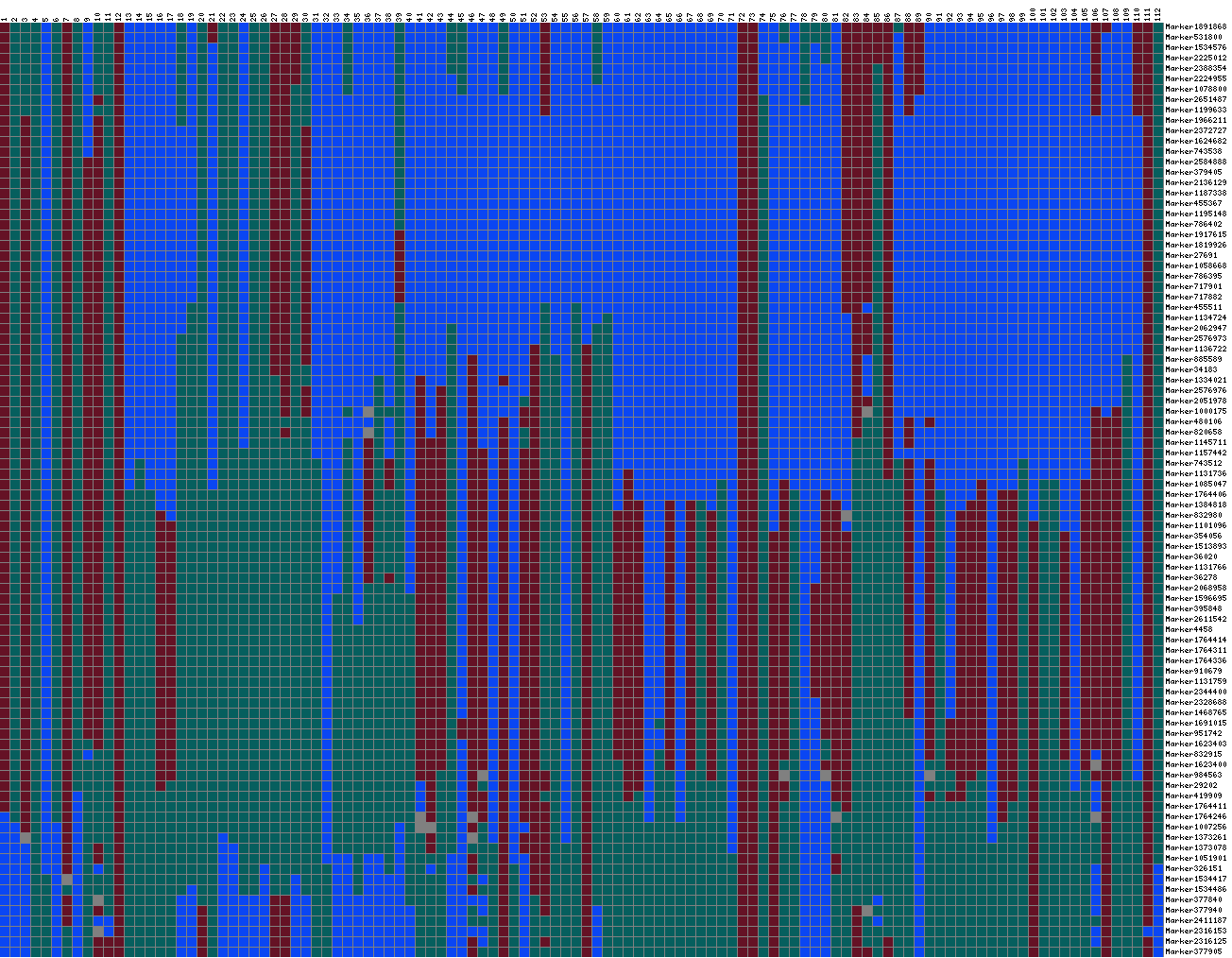

Supplement: Presentation S1 — Haplotype map of the genetic maps. Each row represents a marker. Markers are ranked in accordance with the map order. Each of the two columns represents an individual plant allelic set, with green and blue shading of columns representing the parental alleles. The white column represents that the source of alleles cannot be confirmed. The gray columns represent the deleted alleles. [file Presentation_1.ZIP › supplementary material presentation 1/LG5.haplo.png]

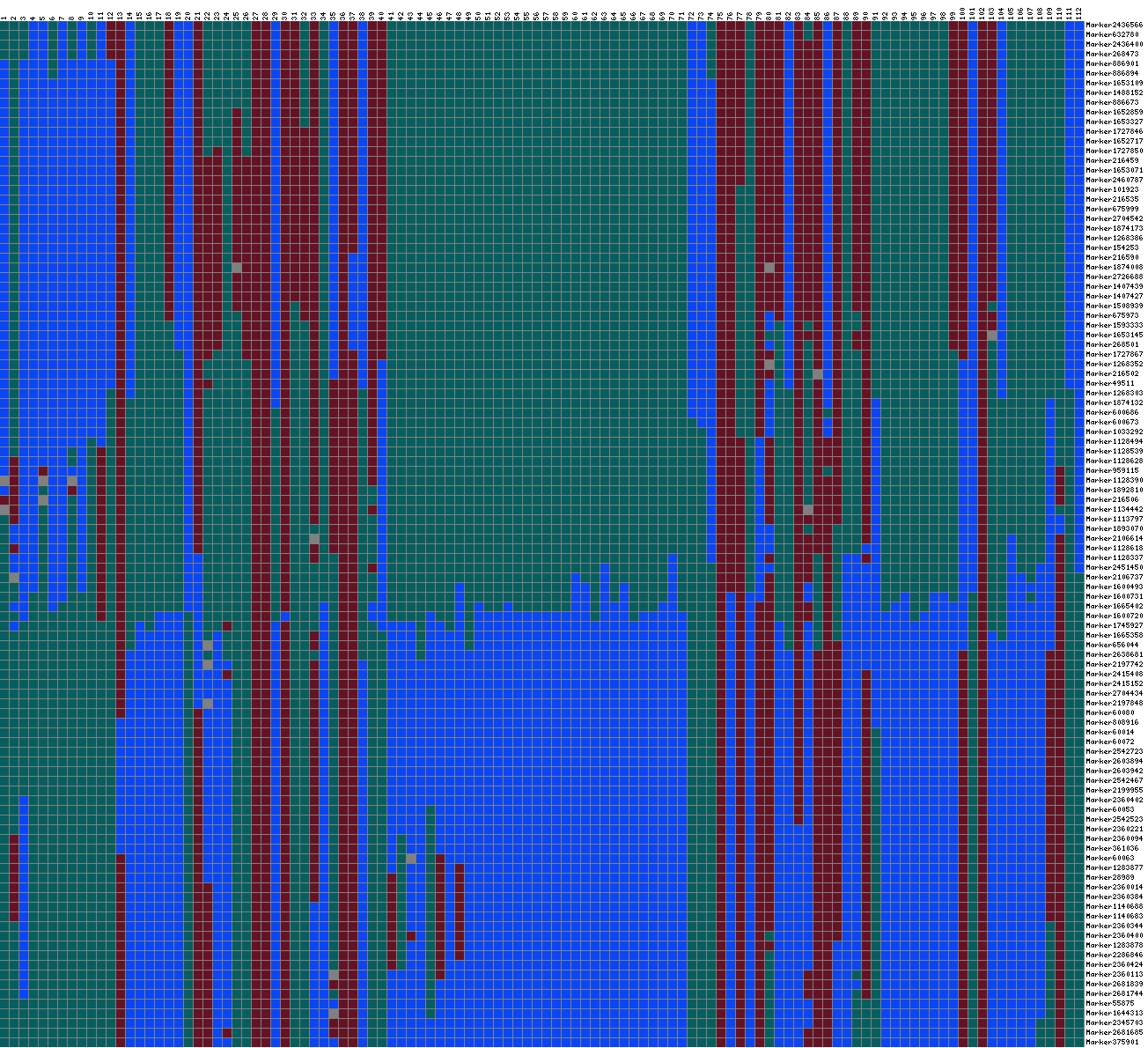

Supplement: Presentation S1 — Haplotype map of the genetic maps. Each row represents a marker. Markers are ranked in accordance with the map order. Each of the two columns represents an individual plant allelic set, with green and blue shading of columns representing the parental alleles. The white column represents that the source of alleles cannot be confirmed. The gray columns represent the deleted alleles. [file Presentation_1.ZIP › supplementary material presentation 1/LG6.haplo.png]

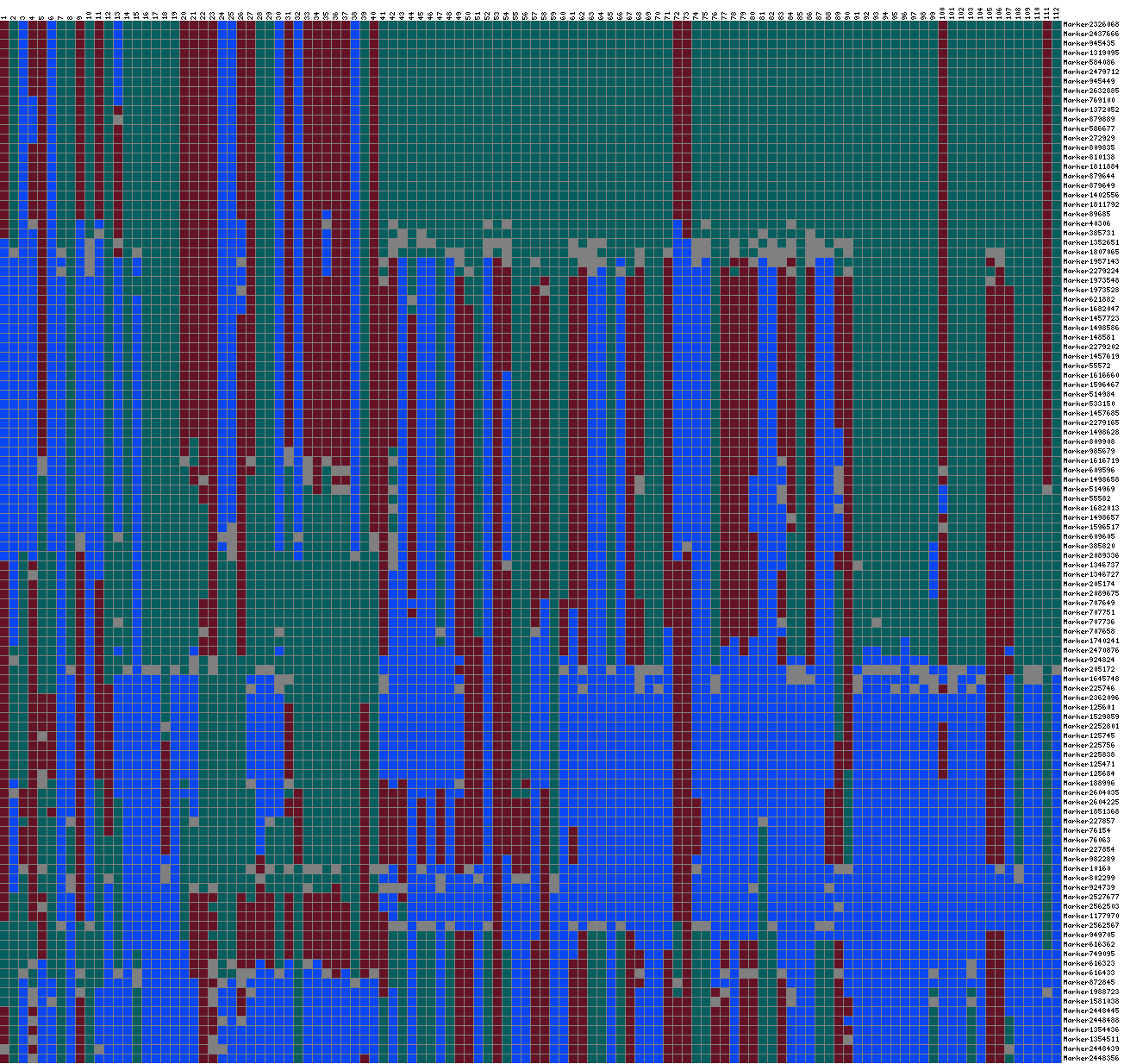

Supplement: Presentation S1 — Haplotype map of the genetic maps. Each row represents a marker. Markers are ranked in accordance with the map order. Each of the two columns represents an individual plant allelic set, with green and blue shading of columns representing the parental alleles. The white column represents that the source of alleles cannot be confirmed. The gray columns represent the deleted alleles. [file Presentation_1.ZIP › supplementary material presentation 1/LG7.haplo.png]

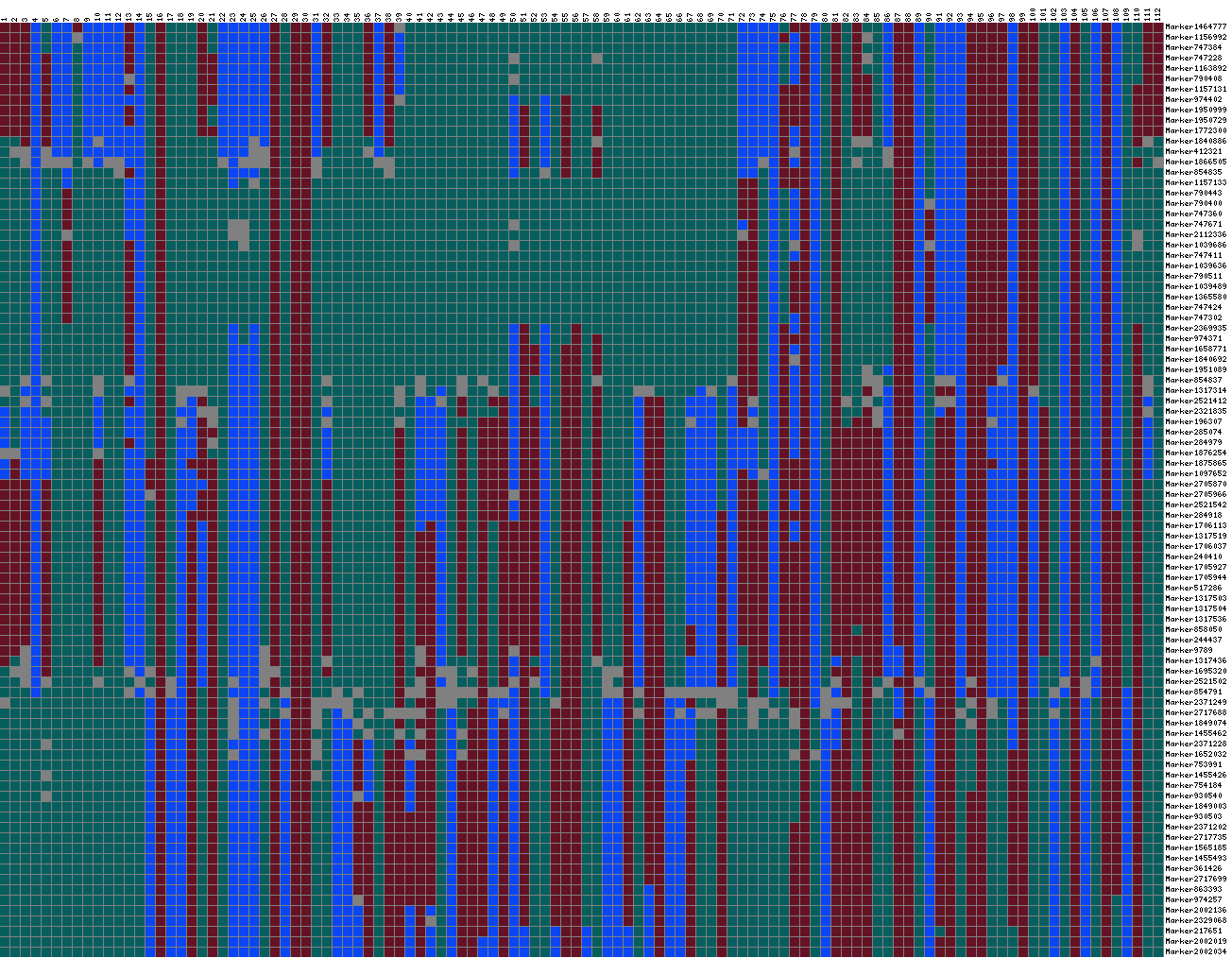

Supplement: Presentation S1 — Haplotype map of the genetic maps. Each row represents a marker. Markers are ranked in accordance with the map order. Each of the two columns represents an individual plant allelic set, with green and blue shading of columns representing the parental alleles. The white column represents that the source of alleles cannot be confirmed. The gray columns represent the deleted alleles. [file Presentation_1.ZIP › supplementary material presentation 1/LG8.haplo.png]

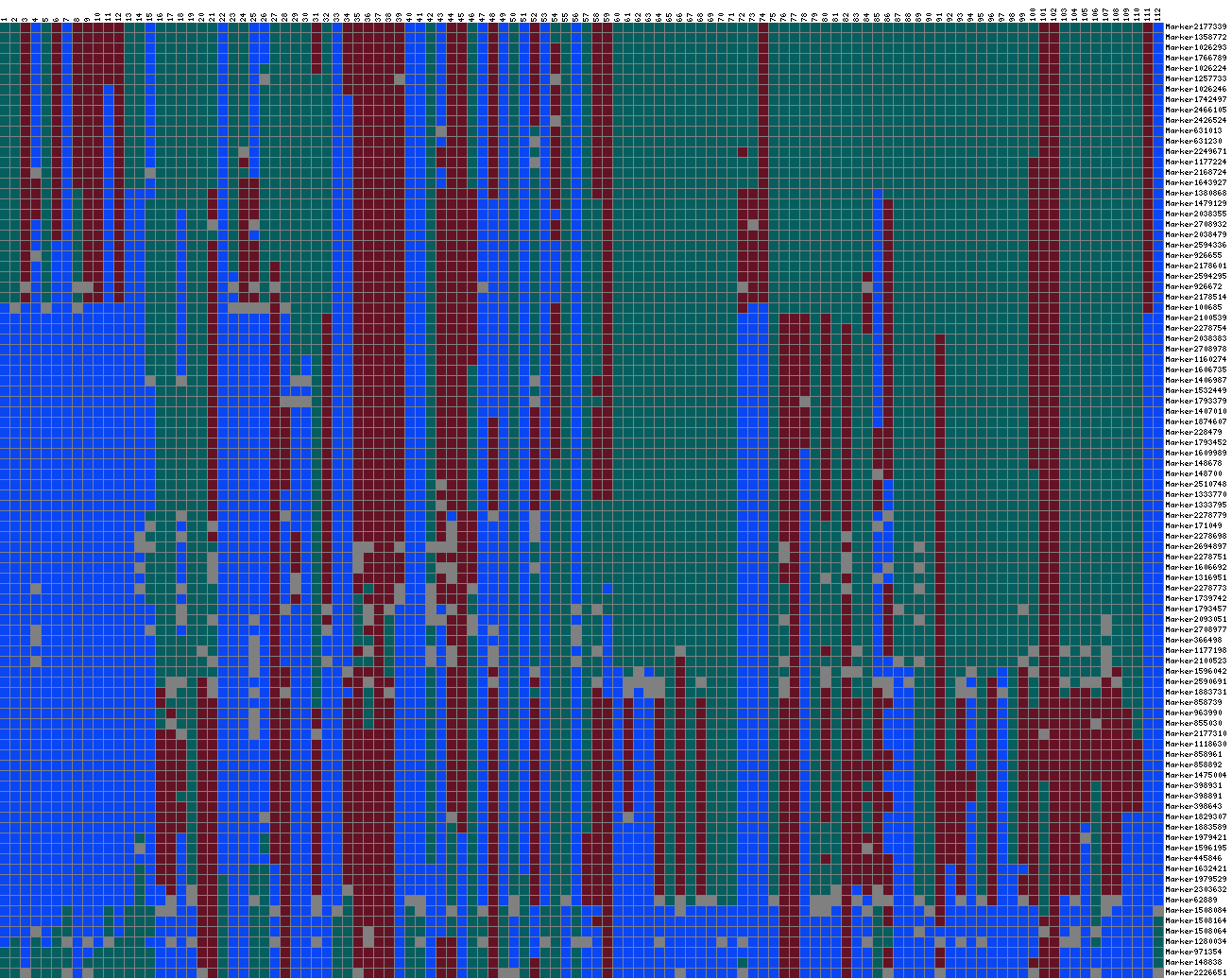

Supplement: Presentation S1 — Haplotype map of the genetic maps. Each row represents a marker. Markers are ranked in accordance with the map order. Each of the two columns represents an individual plant allelic set, with green and blue shading of columns representing the parental alleles. The white column represents that the source of alleles cannot be confirmed. The gray columns represent the deleted alleles. [file Presentation_1.ZIP › supplementary material presentation 1/LG9.haplo.png]

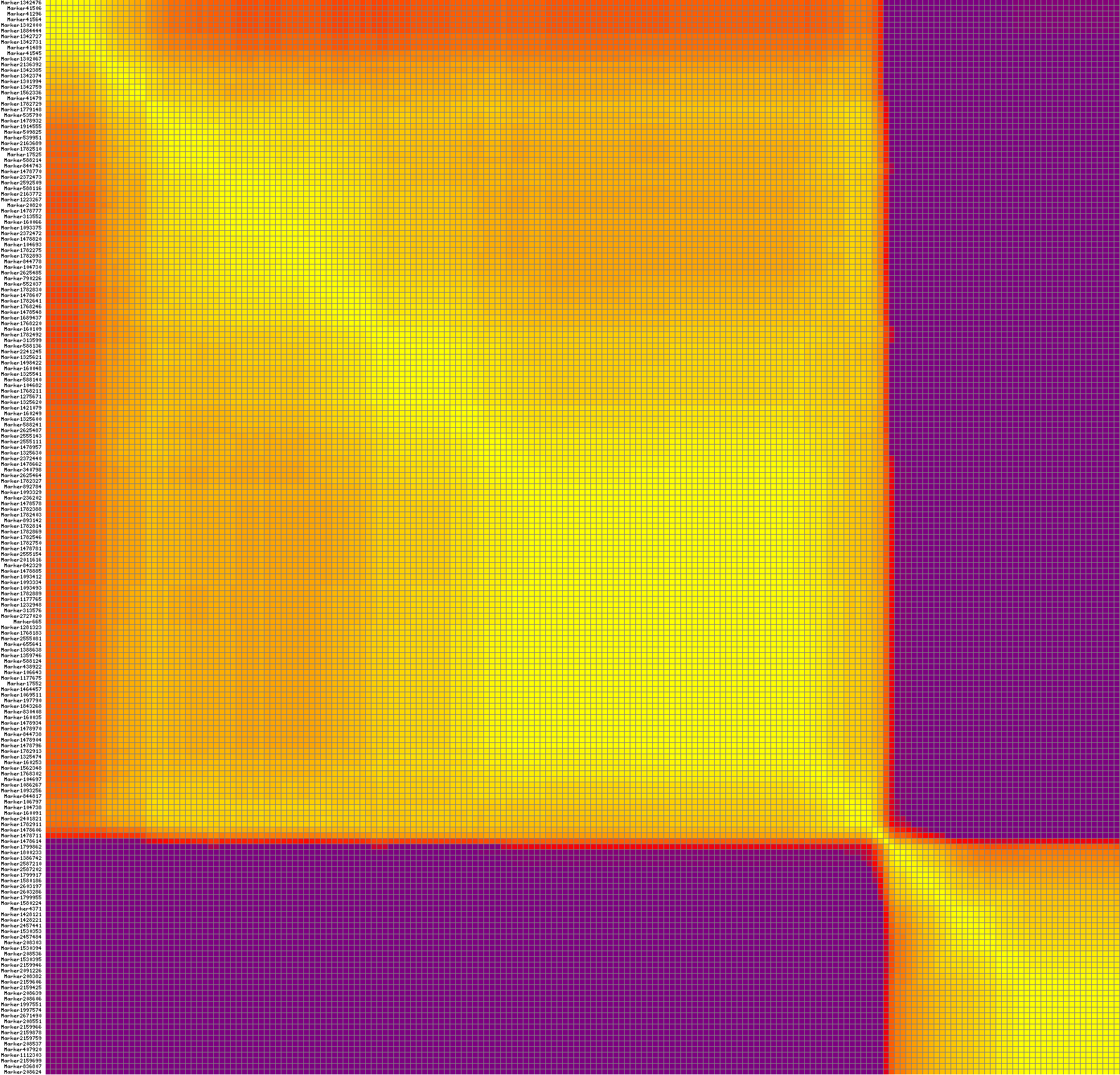

Supplement: Presentation S2 — Heat map of the genetic maps. Markers of each row and column are ranked according to the map order; each small square represents the rate of recombination (r) between the two markers. [file Presentation_2.ZIP › supplementary material presentation 2/LG1.heatMap.png]

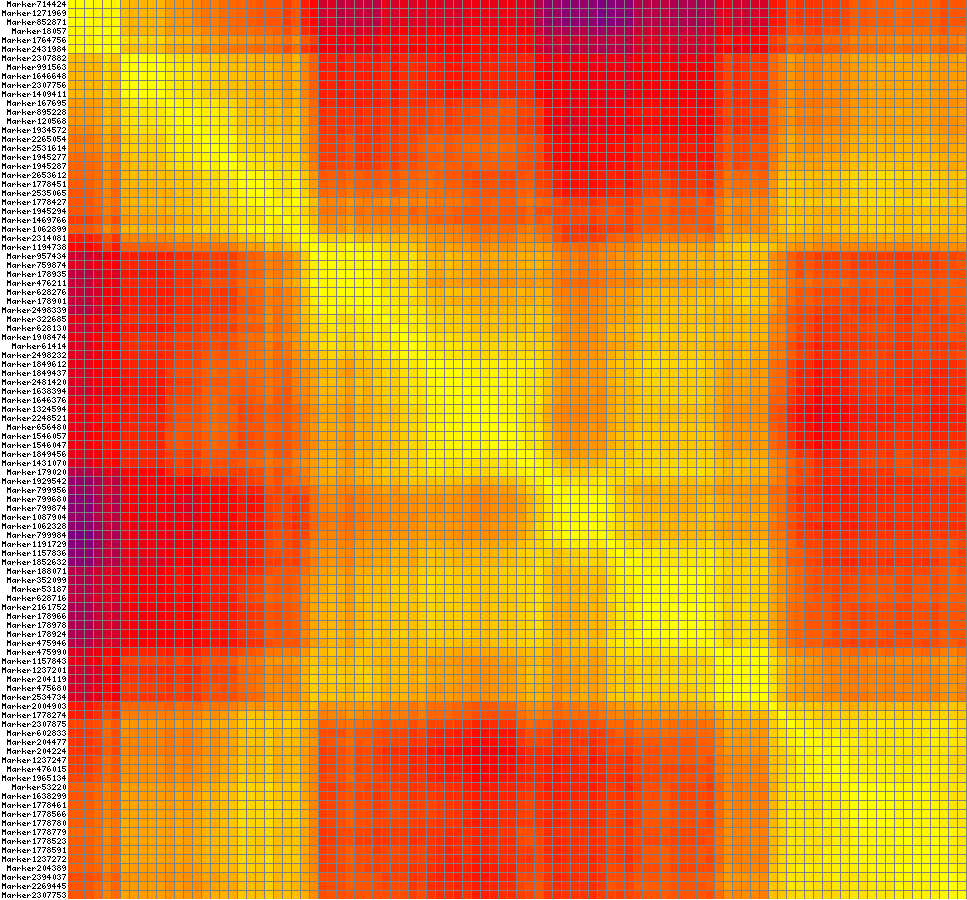

Supplement: Presentation S2 — Heat map of the genetic maps. Markers of each row and column are ranked according to the map order; each small square represents the rate of recombination (r) between the two markers. [file Presentation_2.ZIP › supplementary material presentation 2/LG10.heatMap.png]

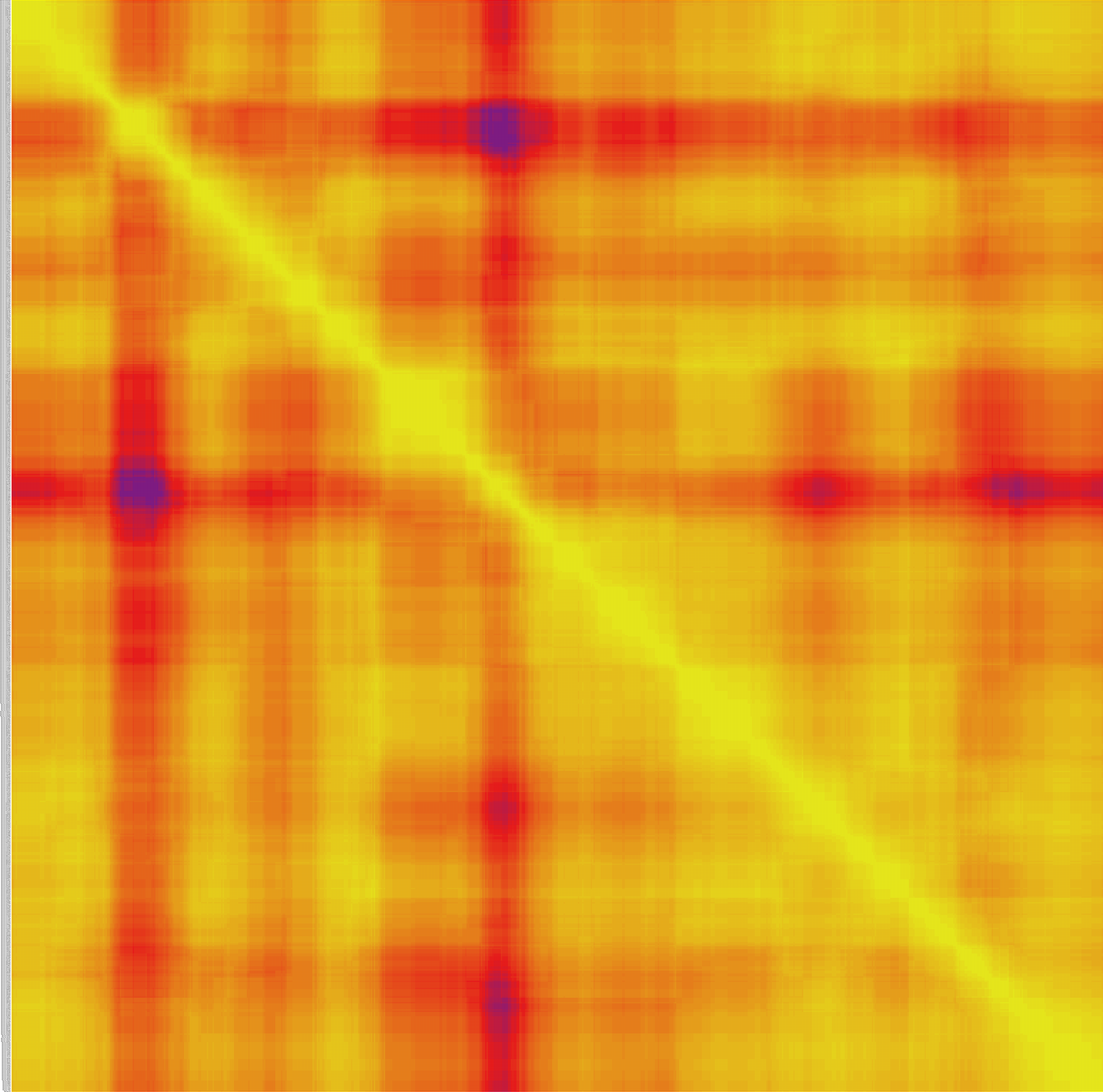

Supplement: Presentation S2 — Heat map of the genetic maps. Markers of each row and column are ranked according to the map order; each small square represents the rate of recombination (r) between the two markers. [file Presentation_2.ZIP › supplementary material presentation 2/LG11.heatMap.png]

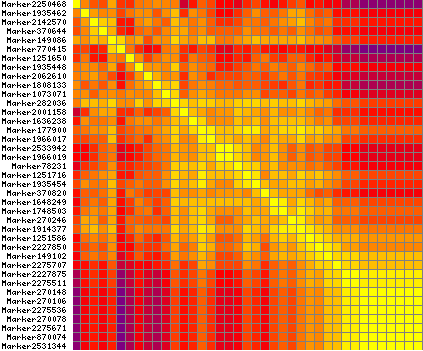

Supplement: Presentation S2 — Heat map of the genetic maps. Markers of each row and column are ranked according to the map order; each small square represents the rate of recombination (r) between the two markers. [file Presentation_2.ZIP › supplementary material presentation 2/LG12.heatMap.png]

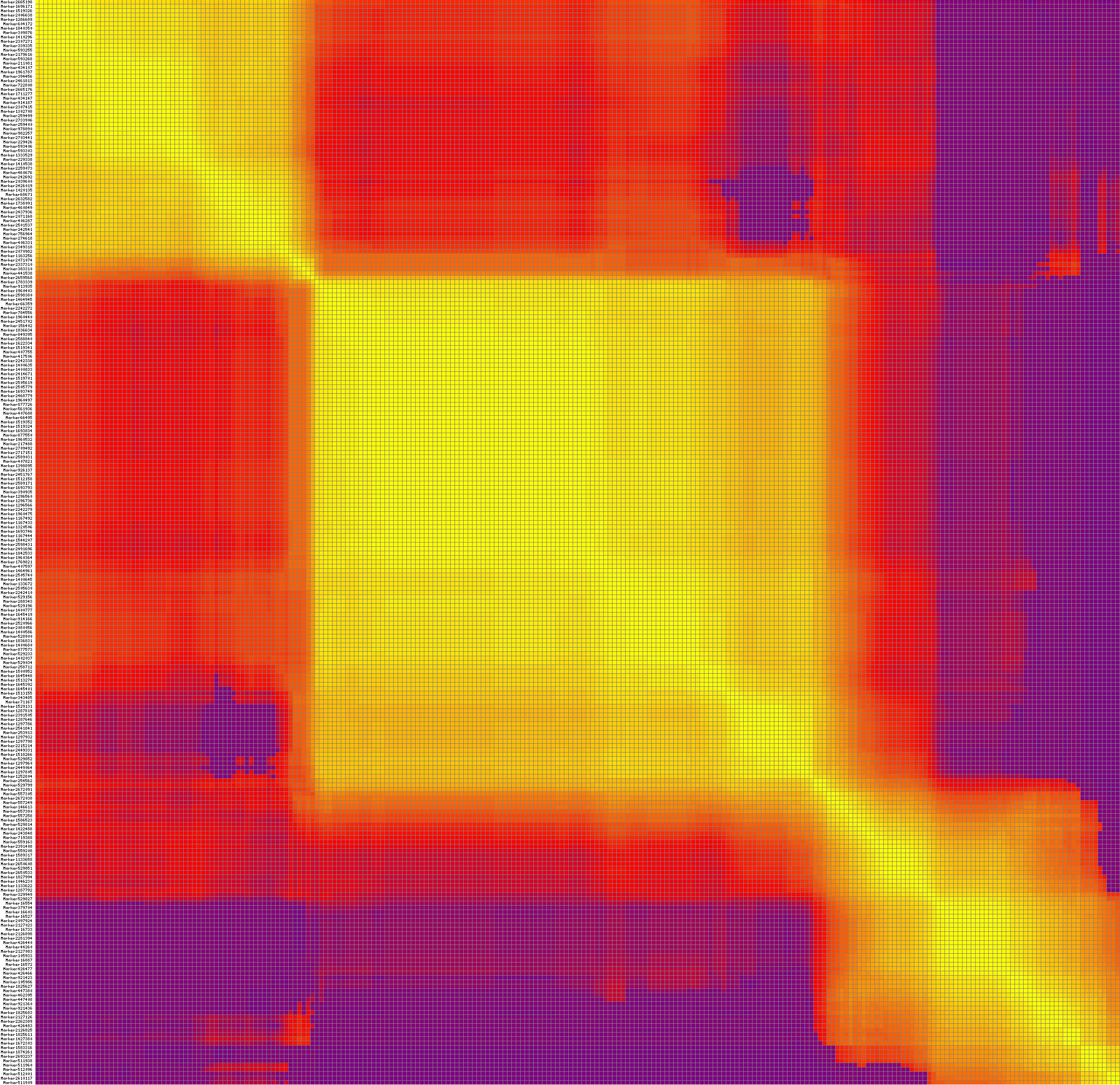

Supplement: Presentation S2 — Heat map of the genetic maps. Markers of each row and column are ranked according to the map order; each small square represents the rate of recombination (r) between the two markers. [file Presentation_2.ZIP › supplementary material presentation 2/LG13.heatMap.png]

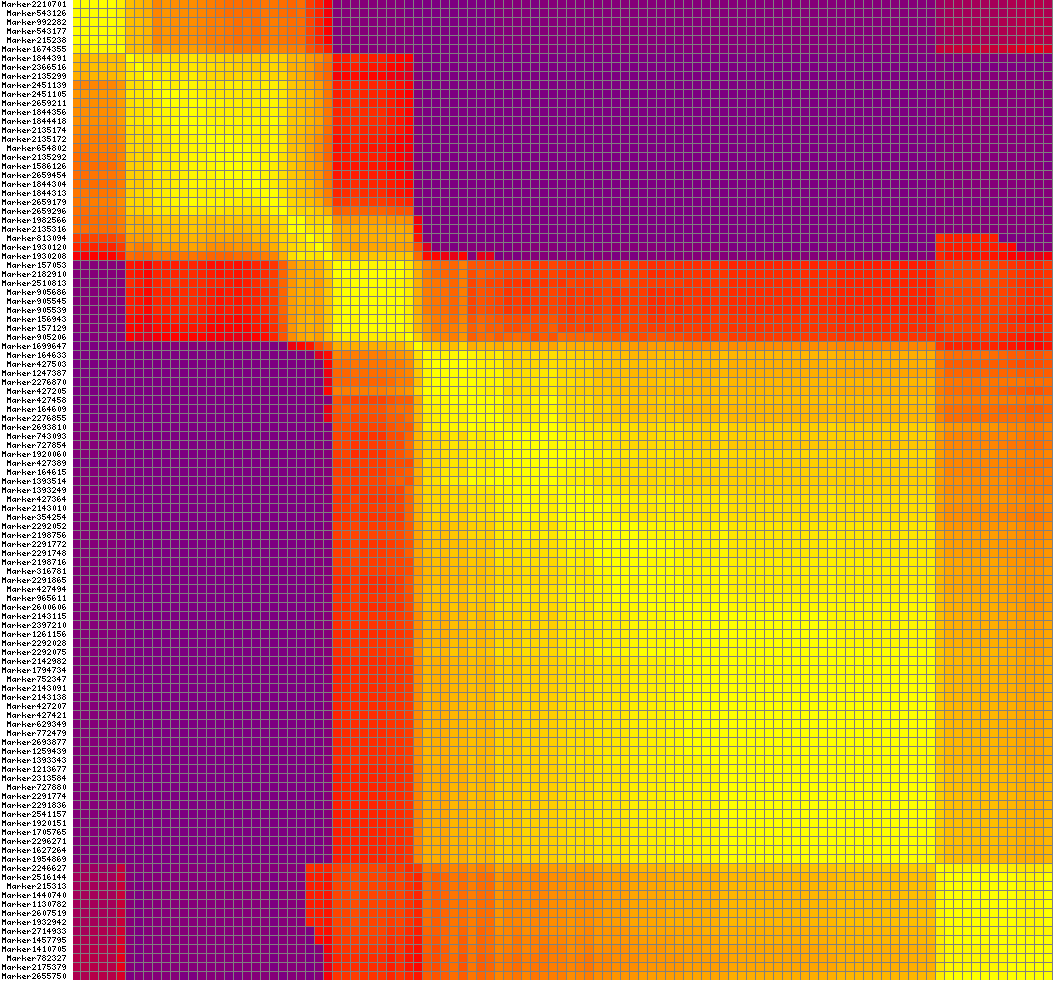

Supplement: Presentation S2 — Heat map of the genetic maps. Markers of each row and column are ranked according to the map order; each small square represents the rate of recombination (r) between the two markers. [file Presentation_2.ZIP › supplementary material presentation 2/LG14.heatMap.png]

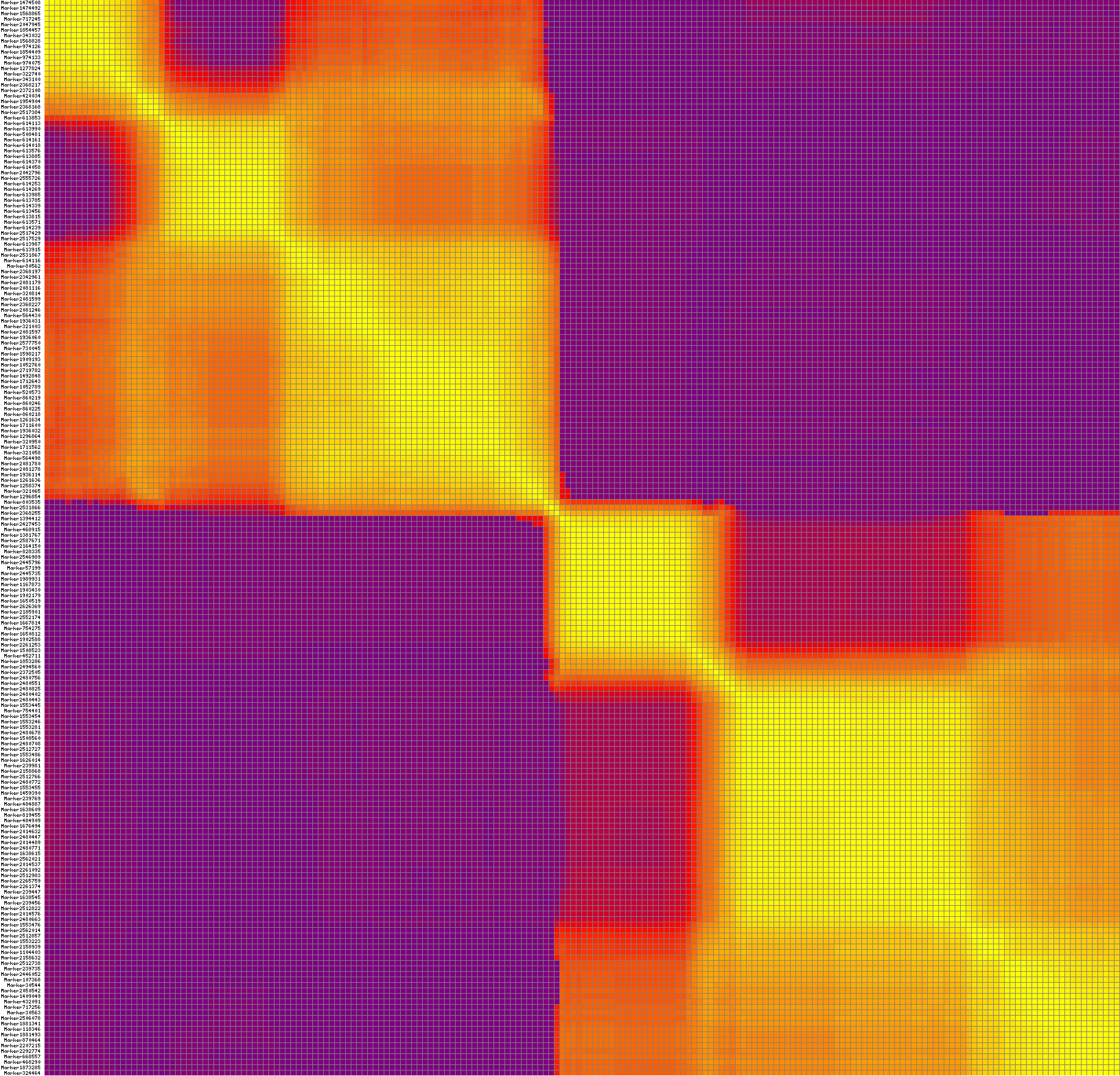

Supplement: Presentation S2 — Heat map of the genetic maps. Markers of each row and column are ranked according to the map order; each small square represents the rate of recombination (r) between the two markers. [file Presentation_2.ZIP › supplementary material presentation 2/LG15.heatMap.png]

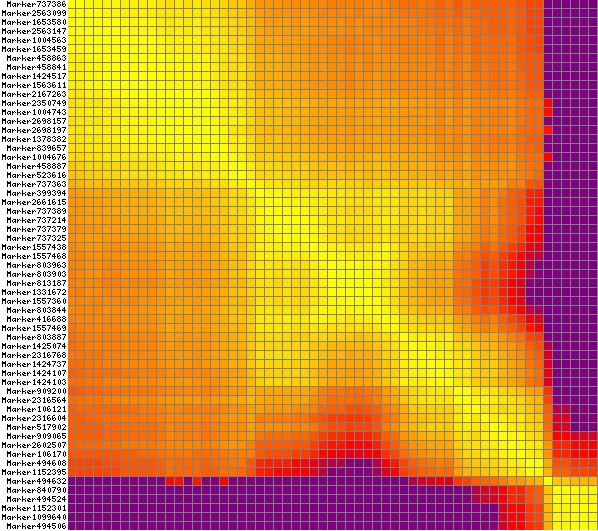

Supplement: Presentation S2 — Heat map of the genetic maps. Markers of each row and column are ranked according to the map order; each small square represents the rate of recombination (r) between the two markers. [file Presentation_2.ZIP › supplementary material presentation 2/LG2.heatMap.png]

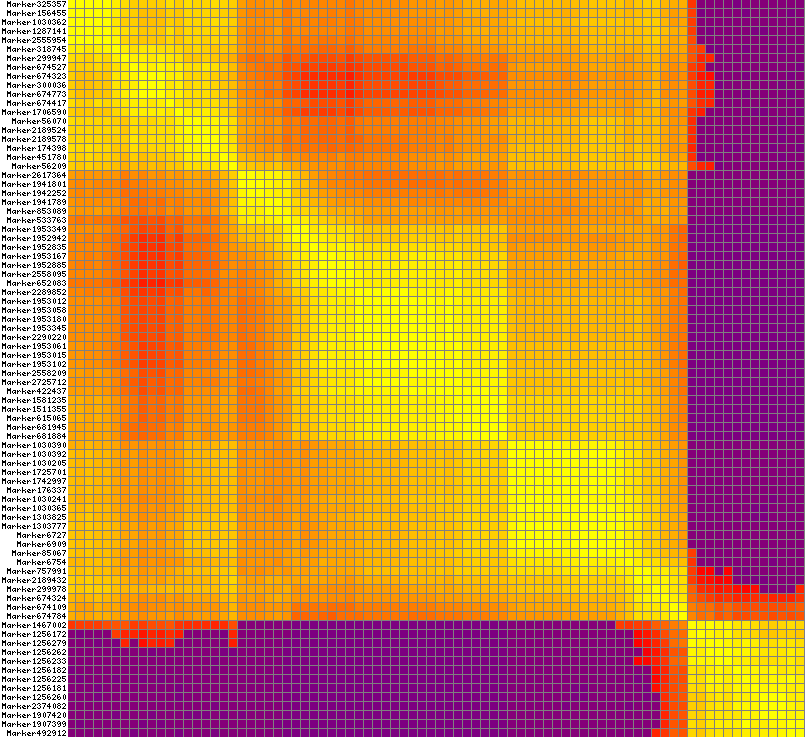

Supplement: Presentation S2 — Heat map of the genetic maps. Markers of each row and column are ranked according to the map order; each small square represents the rate of recombination (r) between the two markers. [file Presentation_2.ZIP › supplementary material presentation 2/LG3.heatMap.png]

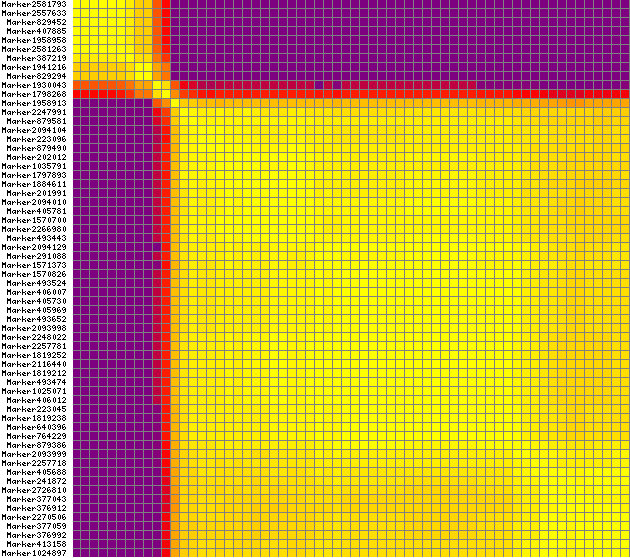

Supplement: Presentation S2 — Heat map of the genetic maps. Markers of each row and column are ranked according to the map order; each small square represents the rate of recombination (r) between the two markers. [file Presentation_2.ZIP › supplementary material presentation 2/LG4.heatMap.png]

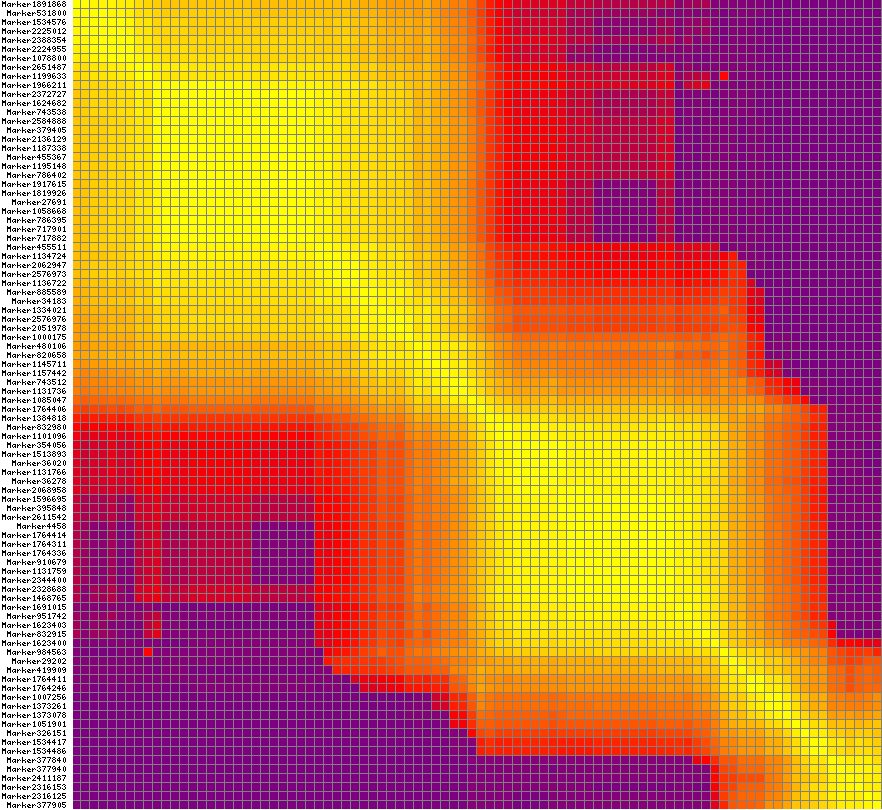

Supplement: Presentation S2 — Heat map of the genetic maps. Markers of each row and column are ranked according to the map order; each small square represents the rate of recombination (r) between the two markers. [file Presentation_2.ZIP › supplementary material presentation 2/LG5.heatMap.png]

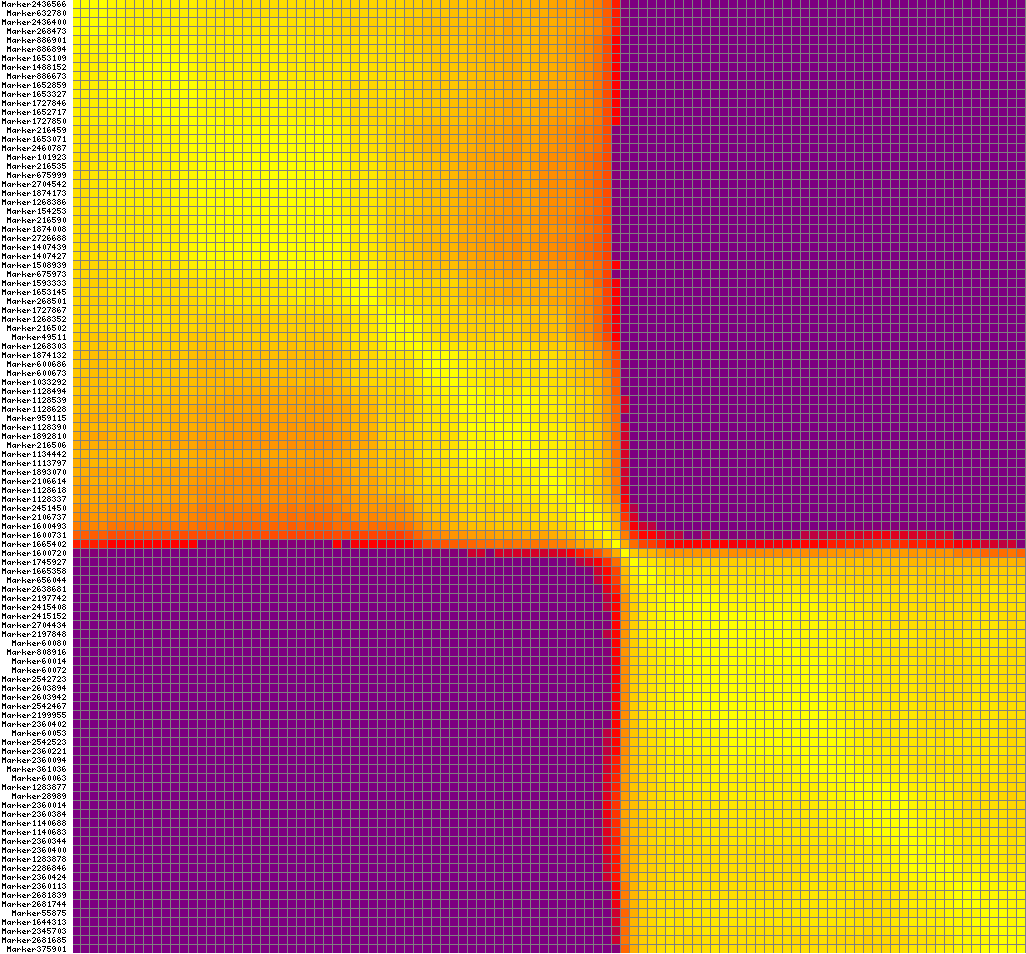

Supplement: Presentation S2 — Heat map of the genetic maps. Markers of each row and column are ranked according to the map order; each small square represents the rate of recombination (r) between the two markers. [file Presentation_2.ZIP › supplementary material presentation 2/LG6.heatMap.png]

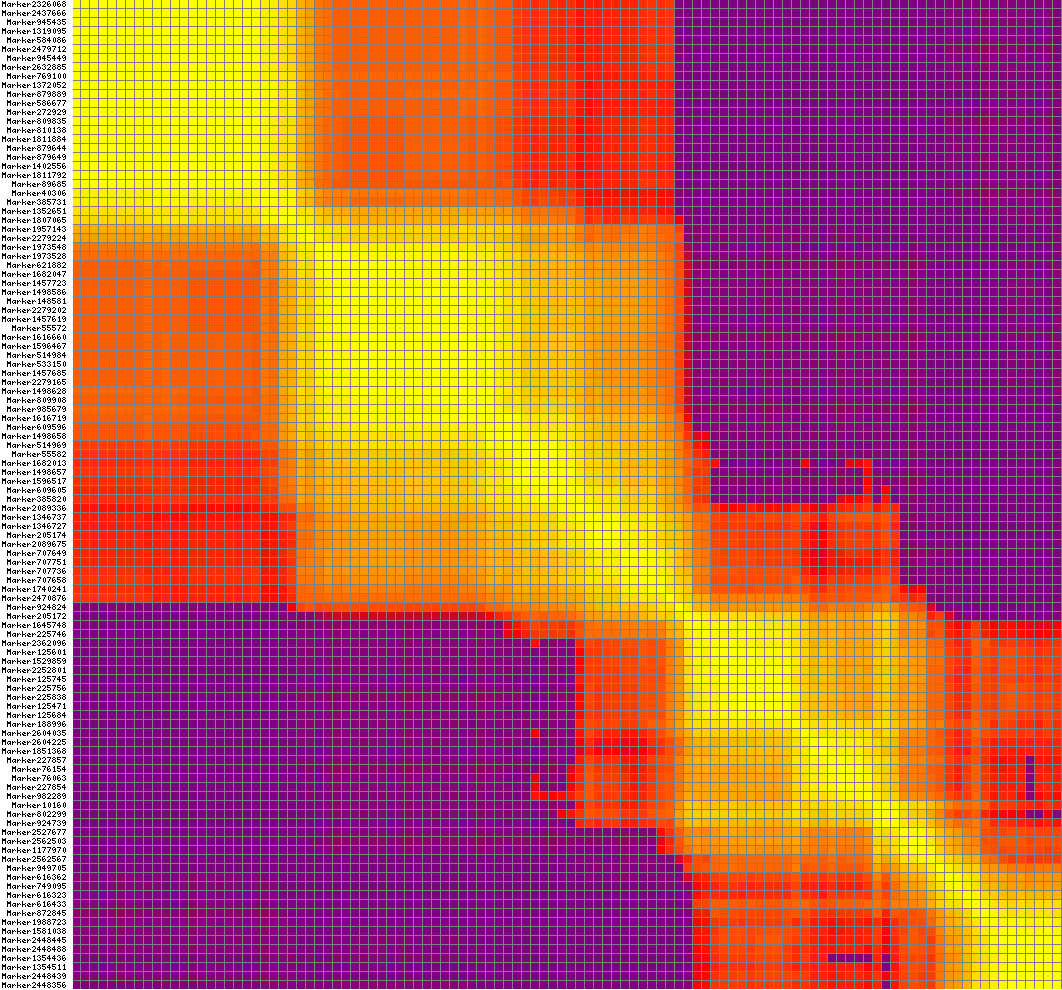

Supplement: Presentation S2 — Heat map of the genetic maps. Markers of each row and column are ranked according to the map order; each small square represents the rate of recombination (r) between the two markers. [file Presentation_2.ZIP › supplementary material presentation 2/LG7.heatMap.png]

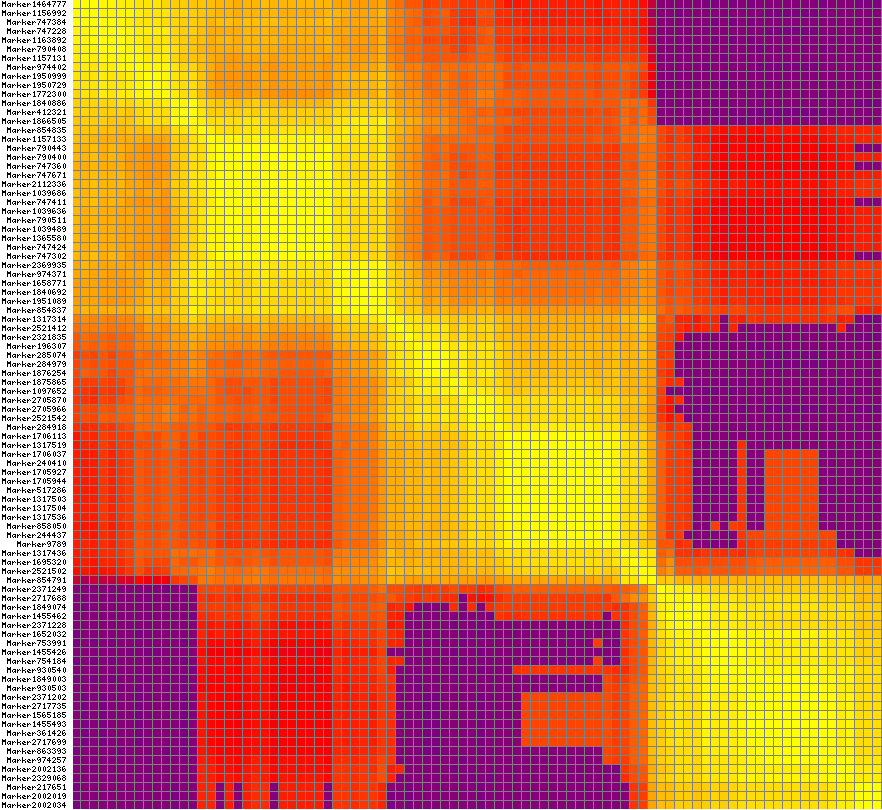

Supplement: Presentation S2 — Heat map of the genetic maps. Markers of each row and column are ranked according to the map order; each small square represents the rate of recombination (r) between the two markers. [file Presentation_2.ZIP › supplementary material presentation 2/LG8.heatMap.png]

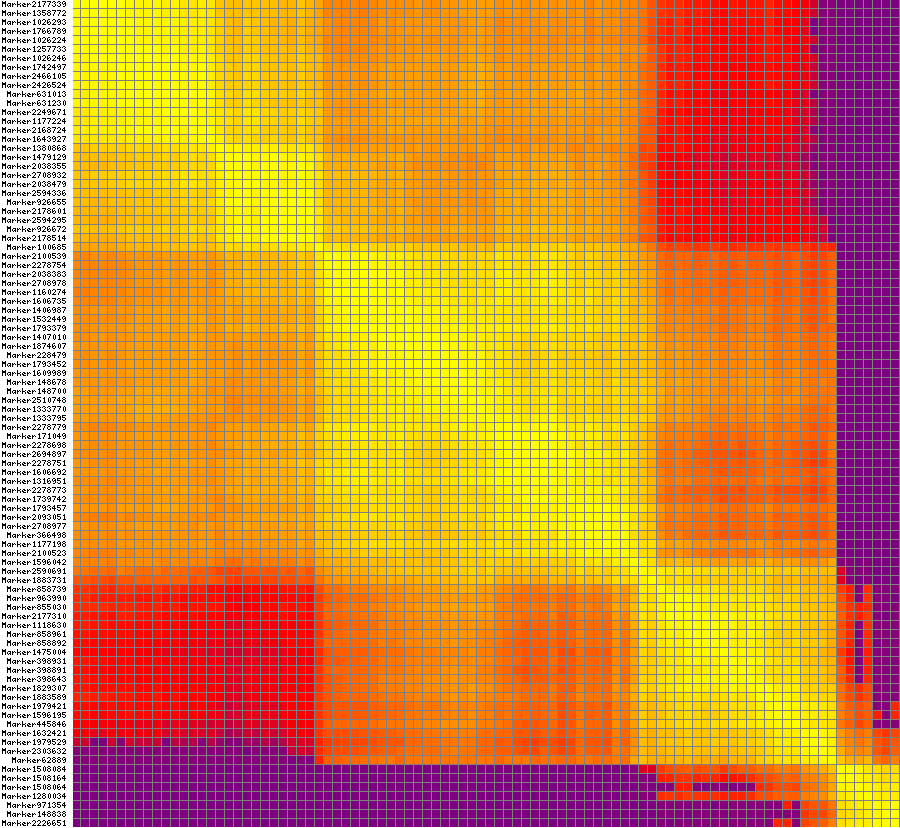

Supplement: Presentation S2 — Heat map of the genetic maps. Markers of each row and column are ranked according to the map order; each small square represents the rate of recombination (r) between the two markers. [file Presentation_2.ZIP › supplementary material presentation 2/LG9.heatMap.png]
